# Out-of-step detection for synchronous generators using electrical power analysis and Durbin Watson testing

**DOI:** 10.1038/s41598-025-03350-8

**Published:** 2025-06-20

**Authors:** R. A. Mahmoud, E. S. Elwakil

**Affiliations:** 1 Department of Electrical Power and Machines Engineering (PME), College of Engineering Science and Technology, Misr University for Science and Technology (MUST), 6th of October City, Giza, Egypt; 2 Power Electronics and Energy Conversion Department, Electronics Research Institute, Cairo, Egypt

**Keywords:** Synchronous generator, Loss of synchronism, Out of step, Unstable power swing, Durbin Watson statistic, Energy science and technology, Engineering

## Abstract

Shunt faults may cause significant fluctuations in the electrical output of the Synchronous Generators (SGs), leading to a loss of synchronization with the remaining power network. Electrical power analysis and the Durbin Watson (DW) statistic can be manipulated to diagnose the instability of the power quality parameters, and to discern between synchronous and asynchronous running of the generator. In this research, the computational techniques serve as a proper foundation of intelligent relay to anticipate and detect the generator Out-of-Step (OOS) situation following the fault presence. The protection strategy can identify sudden variations in several electrical waves in the OOS conditions, such as phase voltage, current, active power, reactive power, and power angle. To verify the performance of the method, a power model with real parameter data of its components is built using the software package of the Alternative Transient Program (ATP). The advanced algorithm is carried out and analyzed using the MATLAB application. Simulation results and analysis show that the protection plan has the ability to recognize the OOS events upon which the protective relay emits a tripping signal to both the annunciation panel and the generator circuit breakers. Whereas, it remains idle under acceptable synchronization conditions. As a consequence, the OOS is rapidly announced before the second pole-slipping occurrence. Furthermore, the algorithm is robust during the stable power swings, and the property of the protection redundancy is provided in this strategy. Additionally, it has the capability of estimating both the instability time and the frequency rate of the unstable power swings.

## Introduction

Synchronous generators (SGs) stability is an important issue in power networks. The SGs must have the same frequency and voltage phase sequence of the connected power network^1^. An Out-of-step (OOS) event of the synchronous generators (SGs) is defined as the out-of-synchronism that is one of the critical and common circumstances, which may result in severe failure to the generator because of stator and rotor overheating^2^. The generated power starts to swing due to faults or disturbances, such as a shunt fault, load shedding, Transmission Line (TL) switching, sudden loss or application of large load block, disconnection of a large generating power plant, and so on. Depending on the severity level of the disturbance, the power fluctuations may not be damped out, which may cause the machine OOS situation (i.e., asynchronous operation)^3^. The OOS phenomenon can damage the power system equipment due to overcurrent and overvoltage effects, or it can result in frequency, voltage, or phase angular instability that may lead to either under or over-frequency incidences, and may cause power grid blackouts^4^. Additionally, when the SG loses a synchronization with the remaining power network, the resulting high peak currents and off-frequency operation may give rise to winding stresses, pulsating torques and mechanical resonance that are potentially deleterious to the turbine-generator^5^. As a consequence, the SG should be isolated as fast as possible during the abnormal conditions to minimize the possibility of the turbine-generator damage and preserve power network stability and service continuity^6^. When the SGs encounter the OOS situations and Out-of-Step Tripping (OST) relays disconnect them from the network, it is popular that adjacent SGs and TLs terminals also face power swings similar to those experienced by the OOS events^7^.

Many protection schemes and systems were presented in the literature for predicting and detecting OOS instants. The impedance-based protection schemes are still operational in power grids, since they are considered as the primary protection against the OOS events^4^. In^5^, an adaptive approach was presented for identifying the OOS by modifying the impedance relay settings. The relay setting was dependent on some parameters of the SG and its connected power grid that should be modified for the setting. Several approaches were dependent on the swing center voltage method^6^, intelligent-based techniques^7,8^, Equal Area Criterion (EAC)^9^, and measuring the angular velocity of the machine and acceleration derived from flux input for expecting and identifying the OOS^10^. Many of the techniques used the Wide-Area Measurement (WAM) systems and units to anticipate and determine the OOS situations in the power grids^11,12^. In^13^, the authors introduced an algorithm based on the concept of a digital phase comparison. The SG terminal phase voltages and currents, besides known generator parameters, were used to estimate the SG internal voltages, where a comparison was carried out between the SG phase terminal and internal voltages to identify OOS. A System Integrity Protection Scheme (SIPS) method was used to adjust protection boundary settings and predict loads^14^. A SIPS design utilizing fault tree analysis and the theory of minimal cut sets is presented to achieve an equilibrium between protection security and dependability^15^. A load-shedding system considering the overload magnitude and time duration was described in^16^, which responds within 0.120 s. Adaptive OOS protection setting according to the power network operating condition improves the security and reliability of this protection was developed in^17^. In^18^, a response-based SIPS scheme was introduced, where two modules were used to maintain system integrity. An apparent impedance-based relay security index (RSI) is the first module to prevent the zone 3 malfunction of impedance relays under severe states and accelerate zone 3 operation during faults. Whereas, the second module is a system stability index (SSI) calculated from the SG rotor angles, which utilized a predictive analysis to foretell a system instability, if any, and take online corrective actions^18^. In^19^, the SG output power and rotor speed were used to predict the OOS. A Synchro-Check Relay (SCR) based on a Pearson correlation was used to satisfy the synchronization conditions for two generators or two segments of a power system^20^. An adaptive OOS technique based on certain SG parameters and power system was used to mend the impedance relay setting in^21^. In^22^, an instability tolerant SG was designed at the power system critical points to decrease power swings and damage. A predictive OOS tripping using synchro-phasors was described in^23^. The technique^23^ was dependent on the phase shifting angle estimated between two nodes in the network or the magnitude of TL current. The method identified the power swing by comparing the swing magnitude with a setting value; after that, a damping factor associated with power swing was used to assess the system stability. The OOS occurrence is eventually a result of unstable power swings when both the damping factor and the computed input signal are greater than the setting value. In^24^, the authors presented coherency determination and stability state for large interconnected power networks based on an analysis of SG frequency responses from several generators simultaneously using online data mining strategy. In^25^, an adaptive technique based on the Bayesian method for predicting the OOS and discriminating between stable and unstable power swings was described. The authors in^13^ proposed an algorithm based on phase comparison for a SGs protection against OOS situations. In^26^, an adaptive tripping index was described for anticipating online OOS occurrence for a Wide Area Measurement Systems (WAMS)-based approach. In^27^, the effect of renewable energy plants on a system transient stability and OOS relay was analyzed, where the relay performance was estimated in definitions of the capability to avoid system instability under distinct abnormal conditions. Dual modules were used in^28^: the first module applied smoothed Pseudo-Wigner–Ville distribution signal processing method to recognize the coherency between the SGs in real-time signal, but the second module used the Lagrangian technique to quickly find the critical SG sets, which were running OOS following severe fault clearance. The scheme in^29^ used both Phasor Measurement Units (PMU) and Equal Area Criterion (EAC), in which SG instability was found by differentiating the acceleration and deceleration areas during the fault time and after fault clearing, respectively. In^30^, the correlation computed between rotor angle/speed oscillations of SGs was applied, where the method concluded a clustering index based on the correlation factors of SG’s oscillations to determine coherent sets of the generators of the power system. In^31^, WAMS-based method and Prony technique were utilized to expect rotor angle instability consisting of transient and small signal. In^32^, the approach used a kinetic energy for estimating the unstable equilibrium point, but this approach is applicable offline as it was dependent on power network modelling. In^33^, the technique harnessed EAC to estimate a SG rotor angle for OOS detection instead of the impedance relay. A PMU installation, global data transmission and related time delay are the major problems in^33^ to adopt the EAC. In^34^, the OOS occurrence was predicted using transient energy calculations. In^35^, the method used a dynamic state estimator for predicting OOS. The SG instability was detected using the state-space model and evaluating Thevenin equivalent in^36^. In^37^, a criterion based on the PMU and the acceleration direction in the critical clearance angle was used for anticipating unstable power swings. In^38^, a set of multiple-criteria decision-making theory and support vector machine classifier in WAMS was applied for instability identification. In^39^, correlation indices calculated for three-phase voltages and currents were used to detect the generator OOS. Online OOS detection was presented using Magnitude-Squared Correlation (MSR) and Magnitude-Squared Coherence (MSC) estimators computed for only the three-phase SG voltages^40^. The computational load of the technique described in^39^ exceeds that of the technique presented in^40^.

The most significant problems of the diverse existing methods for OOS anticipation/detection are given as below.

1. Several approaches take fairly long time to figure out the OOS events^29,33,37^
2. Numerous techniques use complicated mathematical formulations/models for OOS expectation/identification^36,38^
3. Many of the protection schemes are unable to estimate the span of the instability time after predicting the OOS^21^
4. Certain existing methods are active during stable power swings^32,34^
5. Other methods cannot find the OOS instants when the SG runs in the mode of under-excitation^22^
6. Several approaches have lower safety and precision for OOS expectation/detection^13,23^
7. Many different strategies do not apply the stability notion^21,31,38^
8. Some methods work offline^32^
9. Numerous protection plans have a lack of protection redundancy^32,34^
10. Some protection systems have restrictions on a power network size, topology, and the action of power station controllers, encompassing speed governor, automatic voltage regulator, and power system stabilizer^27^
11. A number of impedance-based methods are dependent on the RMS magnitudes of the voltage and current waves, which takes at least a single cycle for calculating the impedance^21^
12. The tripping curves of the traditional impedance relays are based on the parameter specifications of the protected SG, and potential and current transformers^21,26,31,38^
13. Additional low-pass filters are necessary for numerous protection methods^29,33,37^
14. The impedance characteristic settings are constant and without adaptation function according to the operating conditions of the equipment^21,26,29,31,33,37,38^
15. Several Out-of-Step Tripping (OST) relay characteristics only give OST capability, but they cannot provide both OST and Power Swing Blocking (PSB) capabilities^21,26,29,31,33,37,38^
16. The change in the apparent impedance can lead to a malfunction of the impedance-based SG protections if they are not adequately set^21^, and
17. The power network impedance is changed at the SG terminals due to the presence/absence of SG units in a power station, transient events directly affect the machine dynamic parameters, disconnection of power TLs and variation of power network configurations. Therefore, many complexities have been existing for the impedance-based relay due to various relay settings and time-consuming procedure in a multi-machine power system^2,5^

This article presents a new numerical method to detect the generator OOS phenomenon following the temporary/transient faults. The method relies on the electrical power analysis and Durbin Watson (DW) statistic computed for the electrical waveforms of the various variables, such as phase voltages, currents, active and reactive powers, as well as the total active and reactive powers. Monitoring changes in twenty-six DW indices serve as an OOS detector. The discrimination capability between synchronous and asynchronous running of the machine using the Durbin Watson statistic is the main contribution of this paper. The simulation results of various possible test cases demonstrate that the proposed technique is reliable and fast for detecting different types of faults and OOS situations. Furthermore, it can overcome some of the difficulties associated with impedance-based techniques as well.

The sections of this article are organized as follows: the mathematical models and the procedure of the OOS detection are detailed in Section "Proposed methodology". A simulated power grid with real parameter specifications under investigation is shown in Section "Simulated power system under test". Simulation results and analysis of different scenarios are demonstrated in Section "Simulation results and discussion". The salient characteristics of the protection scheme and the major contributions are given in Section "The salient characteristics of the strategy". Finally, the main conclusions of this research are mentioned in Section "Conclusions".

## Proposed methodology

### Algorithm fundamentals

#### Sub-algorithm (1): electrical power analysis

The mathematical model of the total active power (*P*(*k*)) is given as follows^39^:

1 $$P(k) = P_{a} (k) + P_{b} (k) + P_{c} (k)$$

2 $$P(k) = v_{a} (k) \times i_{a} (k) + v_{b} (k) \times i_{b} (k) + v_{c} (k) \times i_{c} (k)$$

whereas, the mathematical model of the total reactive power (*Q(k))* is quantified as follows^39^:

3 $$Q(k) = Q_{a} (k) + Q_{b} (k) + Q_{c} (k)$$

4 $$Q(k) = \frac{1}{\sqrt 3 }\left[ {v_{a} (k) \times \left( {i_{c} (k) - i_{b} (k)} \right) + v_{b} (k) \times \left( {i_{a} (k) - i_{c} (k)} \right) + v_{c} (k) \times \left( {i_{b} (k) - i_{a} (k)} \right)} \right]$$

The quantities (*PF*, *PFA*, *PR*, and *δ*_*s*_) are estimated using the following mathematical equations:

5 $$PF(k) = \cos \left( {\tan^{ - 1} \left( {\frac{Q(k)}{{P(k)}}} \right)} \right)$$

6 $$PFA(k) = \frac{180}{\pi } \times \left( {\tan^{ - 1} \left( {\frac{Q(k)}{{P(k)}}} \right)} \right)$$

7 $$PR(k) = \frac{{\tan^{ - 1} \left( {\frac{Q(k)}{{P(k)}}} \right)}}{{\left| {\tan^{ - 1} \left( {\frac{Q(k)}{{P(k)}}} \right)} \right|}}$$

8 $$V_{sRMS} = \frac{{\sqrt {\sum\nolimits_{k = 1}^{{k = N_{s} }} {\left[ {v_{s} (k)} \right]}^{2} } }}{{N_{s} }}$$

9 $$I_{sRMS} = \frac{{\sqrt {\sum\nolimits_{k = 1}^{{k = N_{s} }} {\left[ {i_{s} (k)} \right]}^{2} } }}{{N_{s} }}$$

The subscript ‘*S*’ represents the phase *A*, *B* or *C*.

10 $$\begin{gathered} P_{a} (k) = v_{a} (k) \times i_{a} (k) = V_{aRNMS} \times I_{aRMS} \times \left[ {\cos (\theta_{a} ) - \cos (2\omega kh - \theta_{a} )} \right] = V_{aRMS} \times I_{aRMS} \times \left[ {\cos (\theta_{a} ) - \cos (\delta_{a} )} \right] \hfill \\ P_{b} (k) = v_{b} (k) \times i_{b} (k) = V_{bRNMS} \times I_{bRMS} \times \left[ {\cos (\theta_{b} ) - \cos (2\omega kh - \theta_{b} - 240^{o} )} \right] = V_{bRMS} \times I_{bRMS} \times \left[ {\cos (\theta_{b} ) - \cos (\delta_{b} )} \right] \hfill \\ P_{c} (k) = v_{c} (k) \times i_{c} (k) = V_{cRNMS} \times I_{cRMS} \times \left[ {\cos (\theta_{c} ) - \cos (2\omega kh - \theta_{c} - 480^{o} )} \right] = V_{cRMS} \times I_{cRMS} \times \left[ {\cos (\theta_{c} ) - \cos (\delta_{c} )} \right] \hfill \\ P_{s} (k) = v_{s} (k) \times i_{s} (k) = V_{sRMS} \times I_{sRMS} \times \left[ {\cos (\theta_{s} ) - \cos (\delta_{s} )} \right] \hfill \\ \end{gathered}$$

11 $$\delta_{S} (k) = \cos^{ - 1} \left[ {\cos (\theta_{s} ) - \frac{{P_{s} (k)}}{{\left( {V_{sRMS} \times I_{sRMS} } \right)}}} \right] = \cos^{ - 1} \left[ {\cos (\theta_{s} ) - \frac{{(2 \times P_{s} (k))}}{{\left( {V_{sMAX} \times I_{sMAX} } \right)}}} \right]$$

The above quantities can be quantified for each data window. The method requires the instantaneous values of the three-phase voltages and currents measured at the load terminals of the synchronous generator.

#### Sub-algorithm (2): Durbin Watson indices

The Durbin Watson (DW) factor is a test statistic used to detect serial correlation in the residuals from a regression analysis^41,42^. A value of DW = 2.0 or nearly 2.0 indicates that there is no first-order autocorrelation. When the value is below 2.0, it indicates a positive serial correlation, and a value higher than 2.0 indicates a negative serial correlation^41,42^.

The DW statistic has the following characteristics^42,43^:

1. It is a dimensionless value; thus, it can be used to compare different variables,
2. It is sensitive to outliers (i.e., non-linear relationship), such as sudden variations in voltage, current, active power, reactive power, frequency, or angular instabilities,
3. Its value is roughly zero when the relationship between two data sets is linear,
4. It is independent of the variations in the measurement scale, and
5. It is considered as a digital low-pass filter. This is because, from signal processing perspective, a smoothing can be achieved using data window concept to minimize variance from short-term variations.

The assumptions of the DW test are as follows:

(I) Errors are normally distributed with a mean value of 0.0, and
(II) All errors are stationary.

The Durbin Watson index (*DWg*_*s*_) can be computed between each two successive data windows that are shifted from each other by a single cycle. Consider the data window distance contains *N*_*w*_ samples of the electrical signal (*g*_*s*_*(k)*) measured/calculated for each ‘*S*’ phase, where ‘*S*’ stands for phase ‘*A*’, ‘*B*’ or ‘*C*’. The Durbin Watson statistic (*DWg*_*s*_) can be defined as^42,43^:

12 $$DWgs = \frac{{\sum\nolimits_{k = 1}^{{N_{w} }} {\left[ {g_{s} (k) - g_{s} (k - N_{s} )} \right]}^{2} }}{{\sum\nolimits_{k = 1}^{{N_{w} }} {\left[ {g_{s} (k)} \right]}^{2} }}$$

The following fourteen DW factors can be calculated using Eq. (12): *DWv*_*a*_, *DWv*_*b*_, *DWv*_*c*_, *DWi*_*a*_, *DWi*_*b*_, *DWi*_*c*_, *DWp*_*a*_, *DWpb*, *DWpc*, *DWq*_*a*_, *DWq*_*b*_, *DWq*_*c*_, *DWp*_*t*_, and *DWq*_*t*_.

Also, the Durbin Watson statistic (*DWg*_*sx*_) can be expressed as^42,43^:

13 $$DWgsx = \frac{{\sum\nolimits_{k = 1}^{{N_{w} }} {\left[ {g_{sx} (k) - g_{sx} (k - N_{s} )} \right]}^{2} }}{{\sum\nolimits_{k = 1}^{{N_{w} }} {\left[ {g_{sx} (k)} \right]}^{2} }}$$

The following twelve DW factors can be computed using Eq. (13): *DWv*_*ab*_, *DWv*_*bc*_*, DWv*_*ca*_*, DWi*_*ab*_*, DWi*_*bc*_*, DWi*_*ca*_*, DWp*_*ab*_*, DWp*_*bc,*_* DWp*_*ca*_*, DWq*_*ab*_*, DWq*_*bc*_, and *DWq*_*ca*_. Where, the wave of *g*_*sx*_(*k*) is the difference between the two waves of *g*_*s*_(*k*) and *gx*(*k*) with the same unit measurment.

14 $$g_{sx} (k) = g_{s} (k) - g_{x} (k)$$

Changes in the DW factors can be used to decide whether to send a trip or restrain signal to the SG circuit breakers. In this concern, it can be applied to detect faults and OOS phenomenon and distinguish between synchronous and asynchronous running of the SG. Test results will prove that the DW algorithm can be used to efficiently identify the fault and loss-of-synchronism conditions for the electrical machine, making the approach is highly reliable and accurate.

### Strategy procedure

To detect the generator OOS situations, the proposed plan requires the following:

1. Measurements of three-phase voltage and current waves at the load terminals of the SG stator windings. Where the three-phase voltages and currents tend to simultaneously sudden variations during the OOS events,
2. Use of the quantities (*PF, PFA*, *PR*, and *δ*_*s*_) derived from the electrical power analysis,
3. Calculation of twenty-six DW factors that confirm sudden changes in electrical variables during the asynchronous operation of the SG, upon which the protective relay sends a tripping signal to the SG breakers, yet it would not act under synchronous and normal operating conditions,
4. After the completion of fault clearance, the DW factors for the three single-phase currents, voltages, active and reactive powers, and the total active and reactive powers are calculated. The unforeseen variations in these factors are monitored. The number of times of the abrupt changes during a predetermined time span (*T*_*os*_) can be counted to monitor the generator OOS, revealing the frequency rate of the power swings. The selected time (*T*_*os*_) is set to 0.5 Sec in this algorithm.

**Fig. 1:**
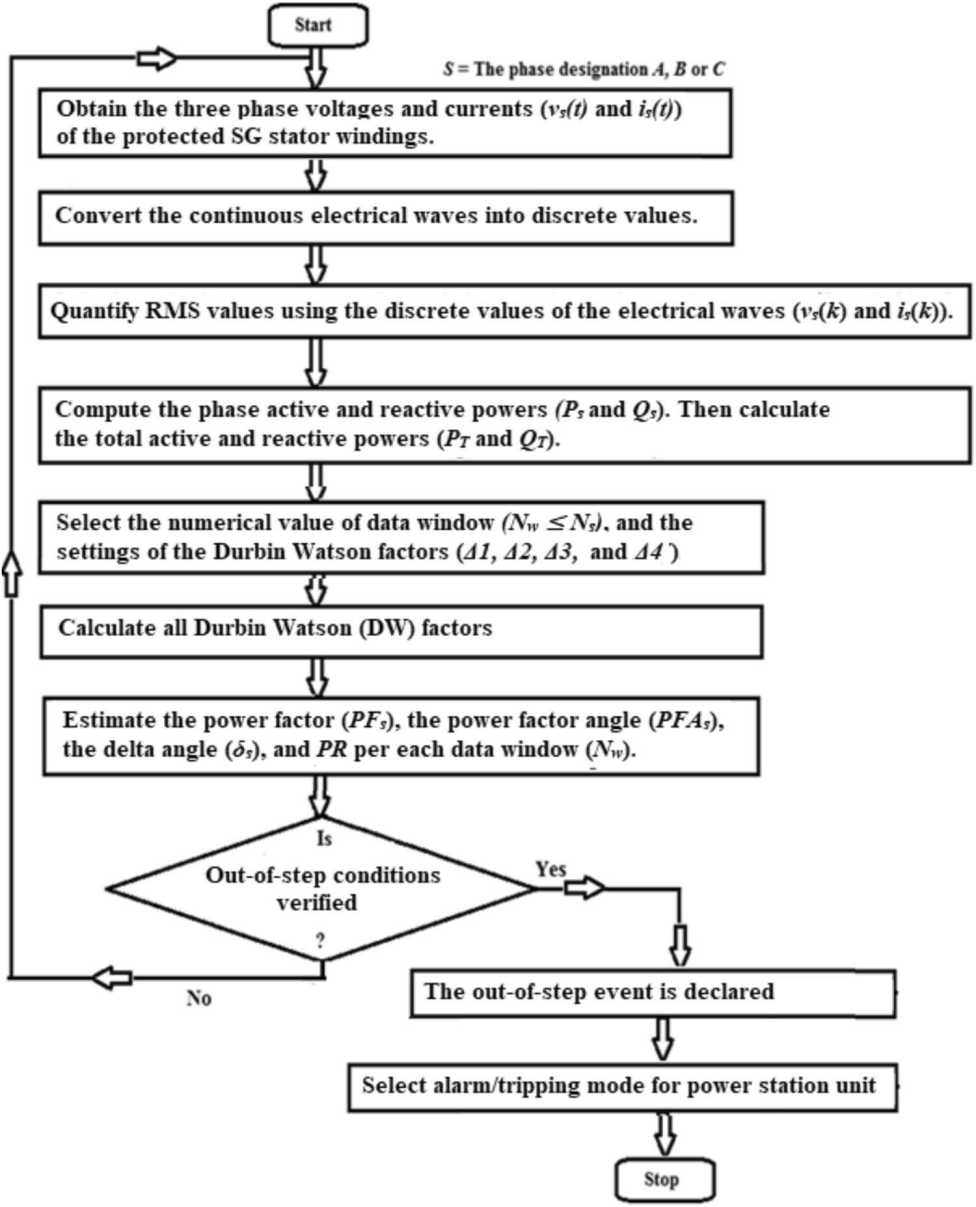
Flowchart for generator out-of-step detection.

Figure 1 illustrates a flowchart for detecting the generator OOS instant. The proposed strategy can be accomplished as follows:

1. Obtain the measurement of the three-phase voltages and currents (*v*_*s*_(*t*) and *i*_*s*_(*t*), respectively) via the potential and current transformers built at the load terminals of the SG stator windings,
2. Transform the continuous electrical waves (*v*_*s*_(*t*) and *i*_*s*_(*t*)) into serial quantities (*v*_*s*_(*k*) and *i*_*s*_(*k*), respectively),
3. Quantify RMS values using the serial values of the electrical signals (*v*_*s*_(*k*) and *i*_*s*_(*k*)),
4. Compute the phase active and reactive powers (*P*_*s*_ and* Q*_*s*_),
5. Calculate the total active and reactive powers (*P* and *Q*),
6. Determine the numerical value of data set (where, *N*_*w*_ ≤ *N*_*s*_), and the DW settings (*Δ*1, *Δ*2, *Δ*3, and *Δ*4),
7. Calculate the twenty-six DW factors noted in Table 1,
8. Estimate the power factor (*PF*), the power factor angle (*PFA*), *PR*, and the delta angle (*δ*_*s*_) for each data set (*N*_*w*_),
9. Specify the zero-crossing positions of the total active power (*P*(*k*)); the zero-crossing point (*k* = *Z*) can be specified using the following condition:
  15 $$T(Z) = P_{{}} (k) \times P_{{}} (k - 1) \le 0.0$$
10. After fault clearing, when the OOS event is achieved according to the given rules in Table 1, the present approach can figure out the OOS situation upon which the relay emits a tripping flag to the SG circuit breakers. Whereas, the output signal is nil under synchronous and normal operating conditions for the SG with the remaining power system.
11. After fault clearance, the following observations can be taken into consideration:
  - The power factor angle (*PFA*) can be obtained for each data set
    (I) In the OOS circumstances, $\theta_{0} < PFA(k)$
      Where, *θ*_0_ is the maximum phase power factor angle permissible, which is set to 50° in the developed algorithm. The operating values of power factor angle (*PFA*) usually lie in the range of 45–90° in the OOS incidences.
    (II) In the instance of the synchronous and normal operation, $\theta_{0} \ge PFA_{{}} (k) \ge 0$
  - The load angle (*δ*_*s*_) can be calculated for each data set
    (I) In the OOS circumstances, $\delta_{0} < \delta_{s} (k)$
      Where,* δ*_*0*_ is the maximum load angle permissible, which is set to 90° in the advanced algorithm. The operating values of the load angle (*δ*_*s*_) are usually located between 90 and 180° in the OOS incidences (i.e., unstable power swings), and their values are within the limit of 50° and 90° in the case of critical stable power swings
    (II) In the instance of the synchronous and normal operation, $\delta_{0} \ge \delta_{s} (k) \ge 0$
    The settings of both *θ*_0_ and *δ*_0_ depend on the prevailing conditions of the generator operation
  - After fault clearance, the first indicator at which *P*(*k*)= *Q*(*k*) is determined. This implies that the *PFA*(*k*) existing between the active and reactive powers is 45^o^ at the sample index ‘*n* = *k*’. After this instant, *Q*(*k*) > *P*(*k*); and the OOS event can be anticipated.
  - After the instant *k* = *n*, the second indicator that is the zero-crossing points at the sample positions *k* = *Z*_1_ and *Z*_2_, at which *P*(*Z*_1_) < 0 and *P*(*Z*_2_) ≥ 0 is identified. Where, *Z*_1_ represents the sample position at which the OOS phenomenon starts, and * Z*_2_ represents the sample position at which the pole-slipping event originates.
  - After fault clearance, the unforeseen changes of the DW indices and the number of occurrence times during the predetermined time interval (*T*_*os*_) will be supervised to detect the OOS circumstances. The predetermined time setting (*T*_*os*_) is 0.5 Sec.
  - When the SG is prone to the OOS incidences or unstable power swings, it will very probably encounter a pole-slipping events for more than once during the time span of 0.5 Sec. It is perfectly unfavorable to allow the SG suffers a pole-slipping for more than once due to a possible severe deterioration to the generator itself and to the rest of power network. Consequently, when the OOS situation is present, the first pole-slipping is declared, before the occurrence of the second pole-slipping, by the protection algorithm.
  - To differentiate between the OOS situations and three phase external shunt faults for the generator, the total active power (*P*) can be monitored consistently to accomplish this function as follows:
    (I) During the OOS events, the value of *P* is less than zero after the fault clearance. Besides, the polarity of *P* wave is transferred from negative to positive, which corresponds to the zero-crossing points that occur simultaneously during the sudden variations of the DW factors.
    (II) Whereas, during the three-phase external faults, the values of *P* wave are greater than or equal to zero. Also, the operating values of the DW factors raise suddenly in the case of three-phase external fault, where these values are larger than those of the OOS phenomenon.
    (III) But, during the synchronous and normal operating conditions, the polarity of *P* wave always remains positive after fault clearing. Furthermore, the operating values of DW factors are nearly constant and close to the DW ideal values, 0.0 or 2.0.
  - The protection sensitivity, security, and response speed are controllable utilizing the data set area (*N*_*w*_) and the DW settings (*Δ*1, *Δ*2, *Δ*3, and *Δ*4).
  - The boundary of the DW settings (*Δ*1, *Δ*2, *Δ*3, and *Δ*4) should be within the two values of 0.0 and 2.0. The DW settings are neatly selected according to the prevailing conditions of the protected machine. In this algorithm, the DW settings (*Δ*1, *Δ*2, *Δ*3, and *Δ*4) are set to 0.15, 2.0, 2.0, and 2.0, respectively.

**Table 1:** Conditions of the synchronous and asynchronous operations for the generator.

Variable/factor	Measured electrical quantity	The value/range of the variable/factor in the case of ideal synchronous operation	Conditions of synchronous operation	Conditions of out-of-step (OOS)
	The numerical values of the Durbin Watson (DW) settings are: *Δ*1 = 0.15, *Δ*2 = 2.0, *Δ*3 = 2.0, and *Δ*4 = 2.0			
Total active power (P)	P(*k*)	P_max_ ≥ P ≥ P_min_	P ≥ 0	P < 0
Total reactive power (Q)	Q(*k*)	Q_max_ ≥ Q ≥ Q_min_	Q ≥ 0	Q < 0
PR	P(*k*) and Q(*k*)	PR = + 1.0	PR = + 1.0	PR = − 1.0
Durbin Watson factor for phase voltage (*DWv*_*s*_(*k*))	*v*_*a*_(*k*) and *v*_*a*_(*k-N*_*s*_)	0.0	0.0 < *DWv*_*a*_ < *Δ*1,	*DWv*_*a*_ ≥ *Δ*1,
	*v*_*b*_(*k*) and * v*_*b*_(*k-N*_*s*_)		0.0 < *DWv*_*b*_ < *Δ*1 and	*DWv*_*b*_ ≥ *Δ*1 or
	*v*_*c*_(*k*) and* v*_*c*_(*k-N*_*s*_)		0.0 < *DWv*_*c*_ < *Δ*1	*DWv*_*c*_ ≥ *Δ*1
Durbin Watson factor for phase current (*DWi*_*s*_(*k*))	*i*_*a*_(*k*) and * i*_*a*_(*k-N*_*s*_)	0.0	0.0 < *DWi*_*a*_ < Δ2,	*DWi*_*a*_ ≥ *Δ*2,
	*i*_*b*_(*k*) and * i*_*b*_(*k-N*_*s*_)		0.0 < *DWi*_*b*_ < *Δ*2 and	*DWi*_*b*_ ≥ *Δ*2 or
	*i*_*c*_(*k*) and* i*_*c*_(*k-N*_*s*_)		0.0 < *DWi*_*c*_ < *Δ*2	*DWi*_*c*_ ≥ *Δ*2
Durbin Watson factor for phase active power (*DWp*_*s*_(*k*))	*P*_*a*_(*k*) and *P*_*a*_(*k-N*_*s*_)	0.0	0.0 < *DWp*_*a*_ < *Δ*3,	*DWp*_*a*_ ≥ *Δ*3,
	*P*_*b*_(*k*) and *P*_*b*_(*k-N*_*s*_)		0.0 < *DWp*_*b*_ < *Δ*3 and	*DWp*_*b*_ ≥ *Δ*3 or
	*P*_*c*_(*k*) and *P*_*c*_(*k-N*_*s*_)		0.0 < *DWp*_*c*_ < *Δ*3	*DWp*_*c*_ ≥ *Δ*3
Durbin Watson factor for phase reactive power (*DWq*_*s*_(*k*))	*Q*_*a*_(*k*) and *Q*_*a*_(*k-N*_*s*_)	0.0	0.0 < *DWq*_*a*_ < *Δ*3,	*DWq*_*a*_ ≥ *Δ*3,
	*Q*_*b*_(*k*) and *Q*_*b*_(*k-N*_*s*_)		0.0 < *DWq*_*b*_ < *Δ*3 and	*DWq*_*b*_ ≥ *Δ*3 or
	*Q*_*c*_(*k*) and* Q*_*c*_(*k-N*_*s*_)		0.0 < *DWq*_*c*_ < *Δ*3	*DWq*_*c*_ ≥ *Δ*3
Durbin Watson factor for total active power (*DWp*_*t*_(*k*))	*P*(*k*) and* P*(*k-N*_*s*_)	0.0	0.0 < *DWp*_*t*_ < Δ4 and	*DWp*_*t*_ ≥ *Δ*4 or
			0.0 < *DWp*_*t*_ < *Δ*4	*DWp*_*t*_ ≥ *Δ*4
Durbin Watson factor for total reactive power (*DWq*_*t*_(*k*))	*Q*(*k*) and * Q*(*k-N*_*s*_) *v*_*a*_(*k*) and* i*_*a*_(*k*)	0.0	0.0 < *DWq*_*t*_ < *Δ*4 and	*DWq*_*t*_ ≥ *Δ*4 or
			0.0 < *DWq*_*t*_ < *Δ*4	*DWq*_*t*_ ≥ *Δ*4
*PFA*_*a*_		*PFA*_*a*_ < 40°,	*PFA*_*a*_ < 90°,	*PFA*_*a*_ ≥ 90°,
*PFA*_*b*_	*v*_*b*_(*k*) and* i*_*b*_(*k*)	*PFA*_*b*_ < 40°, and	*PFA*_*b*_ < 90° and	*PFA*_*b*_ ≥ 90° and
*PFA*_*c*_	*v*_*c*_(*k*) and* i*_*c*_(*k*)	*PFA*_*c*_ < 40°	*PFA*_*c*_ < 90°	*PFA*_*c*_ ≥ 90°
Delta angle (*δ*_*s*_)	*v*_*a*_(*k*) and* i*_*a*_(*k*)	*δ*_*a*_ *< *40°,	*δ*_*a*_ < 50°,	*δ*_*a*_ ≥ 90°,
	*v*_*b*_(*k*) and* i*_*b*_(*k*)	*δ*_*b*_ < 40°, and	*δ*_*b*_ < 50° and	*δ*_*b*_ ≥ 90° and
	*v*_*c*_(*k*) and* i*_*c*_(*k*)	*δ*_*c*_ < 40°	*δ*_*c*_ < 50°	*δ*_*c*_ ≥ 90^o^
Durbin Watson factor for line voltage (*DWv*_*sx*_(*k*))	*v*_*ab*_(*k*) and *v*_*ab*_(*k-N*_*s*_), Where, *v*_*ab*_(*k*) = *v*_*a*_(*k*)–*v*_*b*_(*k*)	0.0	0.0 < *DWv*_*ab*_ < *Δ*1,	*DWv*_*ab*_ ≥ *Δ*1,
	*V*_*bc*_(*k*) and *v*_*bc*_(*k-N*_*s*_), Where, *v*_*bc*_(*k*) = *v*_*b*_(*k*)–*v*_*c*_(*k*)		0.0 < *DWv*_*bc*_ < *Δ*1 and	*DWv*_*bc*_ ≥ *Δ*1 or
	*V*_*ca*_(*k*) and *v*_*ca*_(*k-N*_*s*_), Where, *v*_*ca*_(*k*) = *v*_*c*_(*k*)–*v*_*a*_(*k*)		0.0 < *DWv*_*ca*_ < *Δ*1	*DWv*_*ca*_ ≥ *Δ*1
Durbin Watson factor for line current (*DWi*_*sx*_(*k*))	*i*_*ab*_(*k*) and *i*_*ab*_(*k-N*_*s*_), Where, *i*_*ab*_(*k*) = *i*_*a*_(*k*)–*i*_*b*_(*k*)	0.0	0.0 < *DWi*_*ab*_ < *Δ*2,	*DWi*_*ab*_ ≥ *Δ*2,
	*I*_*bc*_(*k*) and *i*_*bc*_(*k-N*_*s*_), Where, *i*_*bc*_(*k*) = *i*_*b*_(*k*)–*i*_*c*_(*k*)		0.0 < *DWi*_*bc*_ < *Δ*2 and	*DWi*_*bc*_ ≥ *Δ*2 or
	*I*_*ca*_(*k*) and *i*_*ca*_(*k-N*_*s*_), Where, *i*_*ca*_(*k*) = *i*_*c*_(*k*)–*i*_*a*_(*k*)		0.0 < *DWi*_*ca*_ < *Δ*2	*DWi*_*ca*_ ≥ *Δ*2
Durbin Watson factor for difference active power (*DWp*_*sx*_(*k*))	*P*_*ab*_(*k*) and *P*_*ab*_(*k-N*_*s*_), where *P*_*ab*_(*k*) = *P*_*a*_(*k*)–*P*_*b*_(*k*)	0.0	0.0 < *DWp*_*ab*_ < *Δ*3,	*DWp*_*ab*_ ≥ *Δ*3,
	*P*_*bc*_(*k*) and *P*_*bc*_(*k-N*_*s*_), Where, *P*_*bc*_(*k*) = *P*_*b*_(*k*)–*P*_*c*_(*k*)		0.0 < *DWp*_*bc*_ < *Δ*3 and	*DWp*_*bc*_ ≥ *Δ*3 or
	*P*_*ca*_(*k*) and *P*_*ca*_(*k-N*_*s*_), Where, *P*_*ca*_(*k*) = *P*_*c*_(*k*)–*P*_*a*_(*k*)		0.0 < *DWp*_*ca*_ < *Δ*3	*DWp*_*ca*_ ≥ *Δ*3
Durbin Watson factor for difference reactive power (*DWq*_*sx*_(*k*))	*Q*_*ab*_(*k*) and *Q*_*ab*_(*k-N*_*s*_), Where, *Q*_*ab*_(*k*) = *Q*_*a*_(*k*)–*Q*_*b*_(*k*)	0.0	0.0 < *DWq*_*ab*_ < *Δ*3,	*DWq*_*ab*_ ≥ *Δ*3,
	*Q*_*bc*_(*k*) and *Q*_*bc*_(*k-N*_*s*_), Where *Q*_*bc*_(*k*) = *Q*_*b*_(*k*)–*Q*_*c*_(*k*)		0.0 < *DWq*_*bc*_ < *Δ*3 and	*DWq*_*bc*_ ≥ *Δ*3 or
	*Q*_*ca*_(*k*) and *Q*_*ca*_(*k-N*_*s*_), Where, *Q*_*ca*_(*k*) = *Q*_*c*_(*k*)–*Q*_*a*_(*k*)		0.0 < *DWq*_*ca*_ < *Δ*3	*DWq*_*ca*_ ≥ *Δ*3
The action of the protection scheme		Restraining action		Indication/tripping action

## Simulated power system under test

**Fig. 2:**
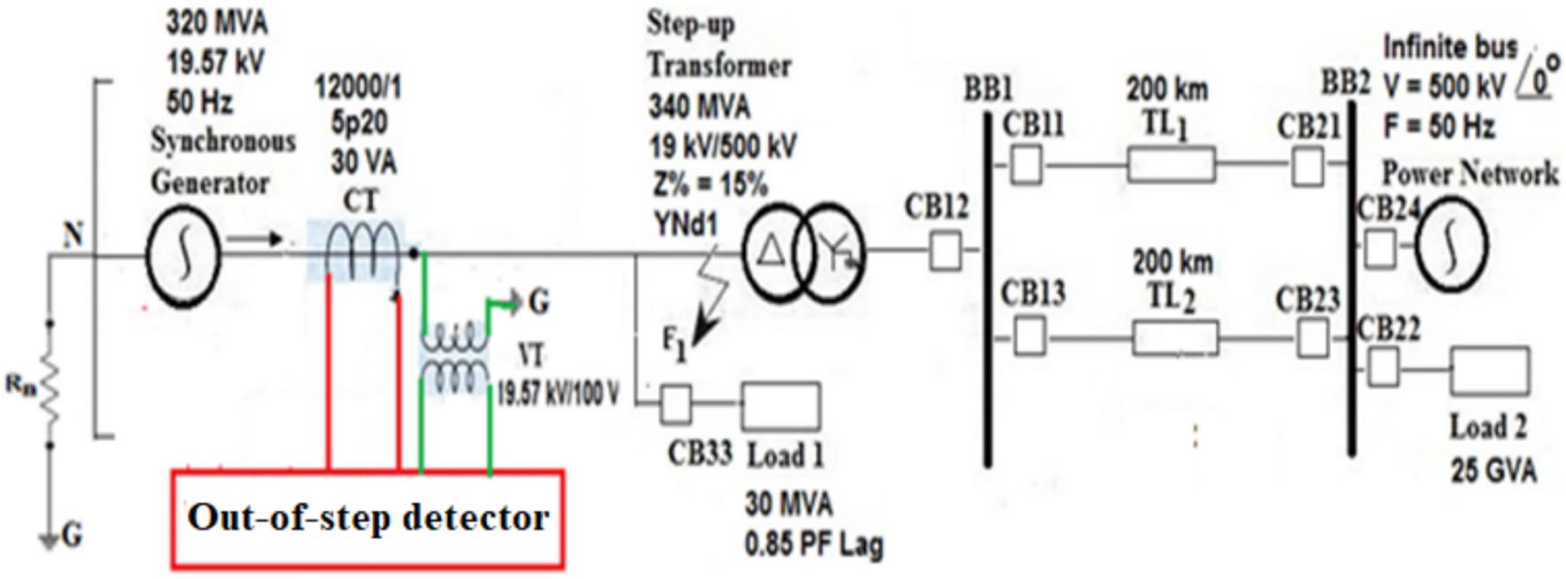
Single line diagram of the power network.

To verify the pertinence of the proposed algorithm, different types of faults are simulated on a typical electrical system using ATP software package. The power system has realistic parameter data^39^. Then, the voltage and current measurements obtained from the simulation cases are processed in MATLAB application program. The application is used to analyze the electrical powers and quantify the Durbin Watson (DW) indices for discriminating between the generator OOS and the acceptable level of synchronization. A single line diagram of the power system under test is illustrated in Fig. 2^39^. The system model consists of a synchronous generator of 320 MVA, 19.57 kV, 50 Hz, a main step-up power transformer of 19.57/500 kV, two busbars of 500 kV, two parallel power transmission lines of 500 kV each 200 km long, and two electrical loads. The first busbar (BB_1_) is existent at the sending end, while the second busbar (BB_2_) is present at the receiving end of the power transmission lines. The first load is located at the terminals of the SG stator windings, while the second load is situated at the second busbar (BB_2_). As shown in Fig. 2, the diagram depicts the potential and current transformers constructed at the SG load ends. The waveforms of voltage and current measured by the instrument transformers serve as input signals to the proposed digital protection. The realistic specifications of the parameters of the system components are given in Appendix 1^39^.

## Simulation results and discussion

The performance of the present technique is estimated through numerous simulation case studies by varying the fault type, fault location, fault resistance, fault inception angle, fault duration time, operating frequency, the load type, and the SG load angle. In this manuscript, the simulation outcomes obtained from only two case studies are extensively discussed. Both scenarios apply DLG (A-B-G) faults that occur at the position of *F*_1_ without any fault resistance (*R*_*f*_). The fault location is situated on the primary side of the generator transformer, as illustrated in Fig. 2. In this study, it is assumed that this fault is temporary and should be automatically cleared. The SG load angle (*δ*_1_) will be 20.5° in case study 1, and it is 21^o^ in case study 2. The system operating conditions during the fault time of each case study are given in Table 2.

**Table 2:** System operating conditions during the fault time of the two case studies.

Case study	Operating load angle (*δ*_*1*_) (in Deg.)	Fault type	Fault location	Fault inception time and fault clearing time (in Sec)	Fault resistance, *R*_*f*_ (in Ω)	The ratios of current and voltage transformers (CTR and VTR)	CT burden, *R*_*b*_ (in Ω)
Case 1: Synchronous operation	20.5^°^	DLG (A-B-G) fault	F_1_ (located at the primary side of the main transformer)	fault inception time (*t*_*f*_)= 0.105 Sec (i.e., the sample index of 525), and the fault clearing time (*t*_*c*_) = 0.225 Sec (i.e., the sample index of 1125)	0.0	CTR = 12 kA/1 A, and VTR = 11 kV/100 V	0.5 + j0.0
Case 2: Out-of-step (OOS)	21^o^						

The measurements of the electrical waves, measured using the ATP software package, are taken at a sampling rate of 100 samples/cycle. The full simulation time (*N*_*sim*_) is 2.8 Sec (i.e., the total number of samples = 14,000 samples). The instant of the fault initiation (*t*_*f*_) is 0.105 Sec, which is equivalent to the sample index (*N*_*f*_) = 525 of the full simulation time. The instant of the fault clearance (*t*_*c*_) is 0.225 Sec, which corresponds to the sample position (*N*_*c*_) = 1125 of the full simulation time. As a result, the fault time interval is *t*_*fd*_ = *t*_*c*_–*t*_*f*_ = 0.120 Sec, which is equal to *N*_*fd*_ = *N*_*c*_–*N*_*f*_ = 600 samples.

Table 3 introduces the pre-fault values for electrical quantities, DW factors and the derived indices of the electrical powers obtained from the simulation results. The results demonstrate how different SG load angles have an effect on the system stability after fault clearance. Furthermore, the SG transient stability is realized using the DW factors and the indices derived from the SG power output, which are estimated before and during the fault time, as well as after fault clearing. The comparative results of the two scenarios reveal the high speed, effectiveness, accuracy, reliability, and robustness of the proposed method over numerous existing methods.

**Table 3:** Pre-fault values for electrical variables, DW factors and deduced indices of electrical powers obtained from the simulation results.

Parameter data	Case 1	Case 2
Generator status	Synchronous operation state	Out-of-step state
Operating power angles	*δ*_1_ = 20.5°	*δ*_1_ = 21°
	*δ*_2_ = 0°	*δ*_2_ = 0°
*v*_*smax*_ (*kV*)	16.06	16.06
*i*_*smax*_ (*kA*)	8.45	8.53
*P*_*smax*_ (*MW*)	129.5	130.435
*Q*_*smax*_ (*MW*)	96.56	97.59
*P* (*MW*)	184.8	185
*Q* (*MVAR*)	85.96	87.41
*PFA* (*Deg*)	24.96°	25.18°
*PF*	0.906	0.905
*PR*	+ 1.0	+ 1.0
*DWi*_*s*_	0.0	0.0
*DWi*_*sx*_	0.0	0.0
*DWp*_*s*_	0.0	0.0
*DWp*_*sx*_	0.0	0.0
*DWq*_*s*_	0.0	0.0
*DWq*_*sx*_	0.0	0.0
*DWv*_*s*_	0.0	0.0
*DWv*_*sx*_	0.0	0.0
*DWpt*	0.0	0.0
*DWqt*	0.0	0.0

### Synchronous and normal operating condition (***δ***_1_ = 20.5°)

**Fig. 3:**
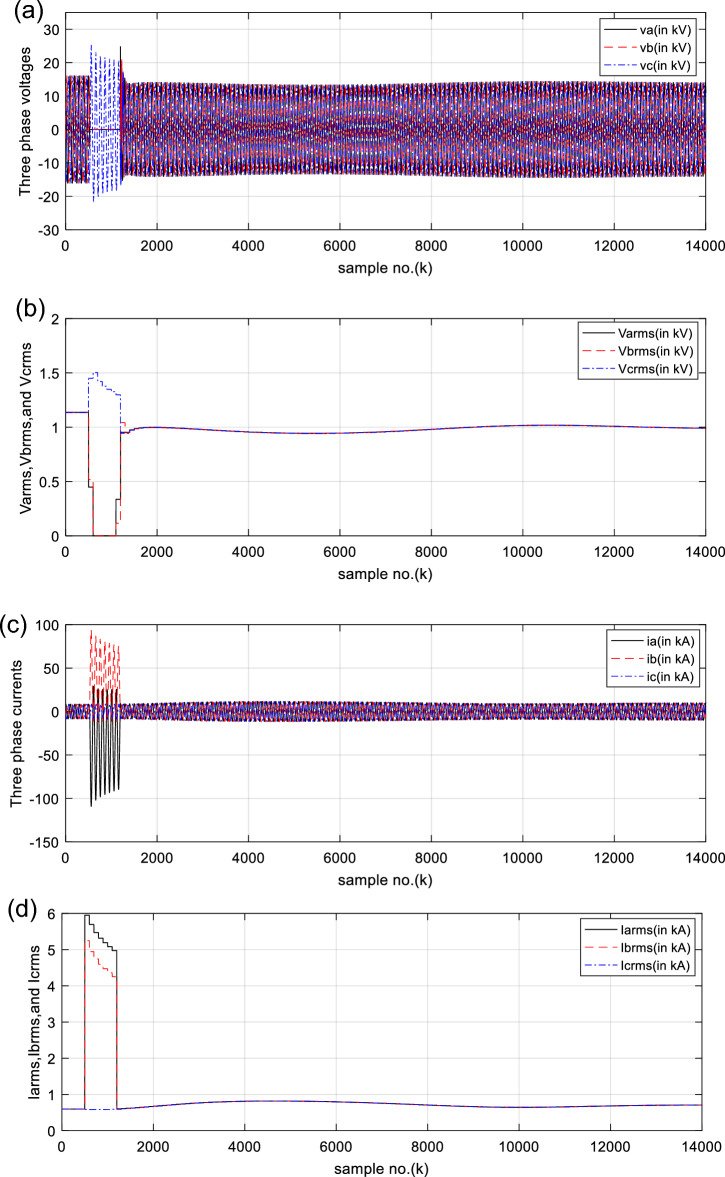
(**a**) Three-phase primary voltage waves measured taken at the SG load terminals. (**b**) RMS values of three-phase primary voltage waves taken at the SG load terminals. (**c**) Three-phase primary current waves taken at the SG load terminals. (**d**) RMS values of three-phase primary current waves taken at the SG load terminals. (**a**–**d**) Electrical waveforms for case 1.

**Fig. 4:**
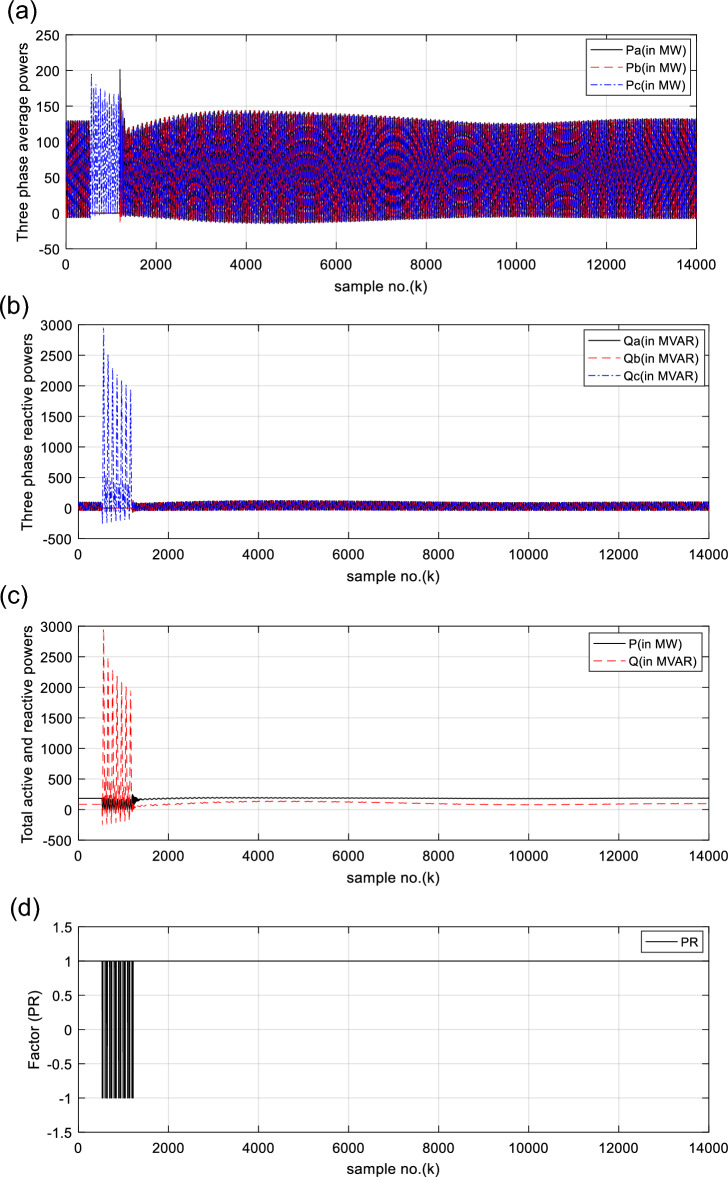
(**a**) Three-phase primary average power waves taken at the SG load terminals. (**b**) Three-phase primary reactive power waves taken at the SG load terminals. (**c**) Total primary average and reactive powers at taken the SG load terminals. (**d**) *PR* factor for case 1. (**a**–**d**) Electrical waveforms and *PR* factor for case 1.

In this verification, the SG load angle is *δ*_1_ = 20.5°, while the network load angle is *δ*_2_ = 0°. The system operating conditions during the time of *DLG* (*A-B-G*) fault are given in Table 2. The simulation results assert that the SG is operational and synchronized with the remaining power grid before the fault initiation and after the fault clearance. As shown in Figs. 3 and 4, the values of the pre-fault voltage, currents, active and reactive powers for the three-phase SG are stationary. Figure 3a and b present the three-phase primary voltage waves taken at the SG load terminals and their RMS quantities, respectively. Figure 3c and d introduce the three-phase primary current waves obtained at SG load ends and their RMS quantities, respectively.

The curves of the three single-phase primary active and reactive powers of the generator are shown in Fig. 4a and b, respectively. Figure 4c demonstrates the two waves of the total primary active and reactive powers (*P* and *Q*) of the generator, where their quantities are greater than zero after fault clearing. Figure 4d depicts the *PR* factor in the case of synchronous operation, where its values are + 1.0 after fault clearing. After fault clearing, the simulation results indicate the synchronous operation of the SG with the rest of power grid.

Table 3 lists the pre-fault quantitative findings of the electrical quantities, DW factors and deduced indices obtained from the simulation results for case 1. During the fault, the voltages of the two faulty phases (*A* and *B*) decrease, while their currents increase, as depicted in Fig. 3a–d, respectively. Besides, the active and reactive powers of the two faulty phases vanish, as depicted in Fig. 4a and b, respectively. These variations correspond with the SG transient stability notion. After fault clearing, the SG under test is synchronous with the connected power grid.

**Fig. 5:**
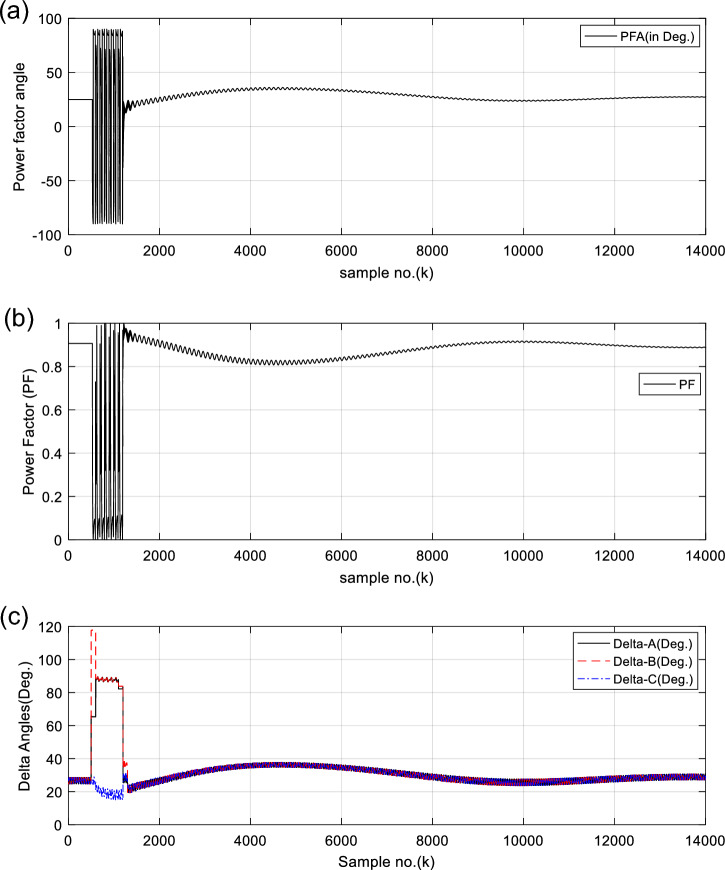
(**a**) Power factor angle (*PFA*) for case 1. (**b**) Power factor (PF) for case 1. (**c**) Computed power angles (δs) for three phases for case 1. (**a**–**c**) PFA, PF, Delta-A, Delta-B, and Delta-C for case 1.

Figure 5a and b offer the power factor angle (*PFA*) and the power factor (*PF*) in the case of synchronous operation of the generator, respectively. In this case, the values of the power factor (*PF*) vary within 0.80 and 0.91 after fault clearance. To increase the redundancy of the generator OOS protection, the SG load angles (*δ*_*s*_) are computed for each data window per each phase. Figure 5c presents the computed load angles (*δ*_*s*_) for three single-phases of the SG in case study 1. After fault clearance, the load angles (*δ*_*a*_*, δ*_*b*_*,* and* δ*_*c*_) change from 21.3 to 37.8°, as depicted in Fig. 5c. These angles confirm that the power swings are stable and the SG is in sync with the system.

**Fig. 6:**
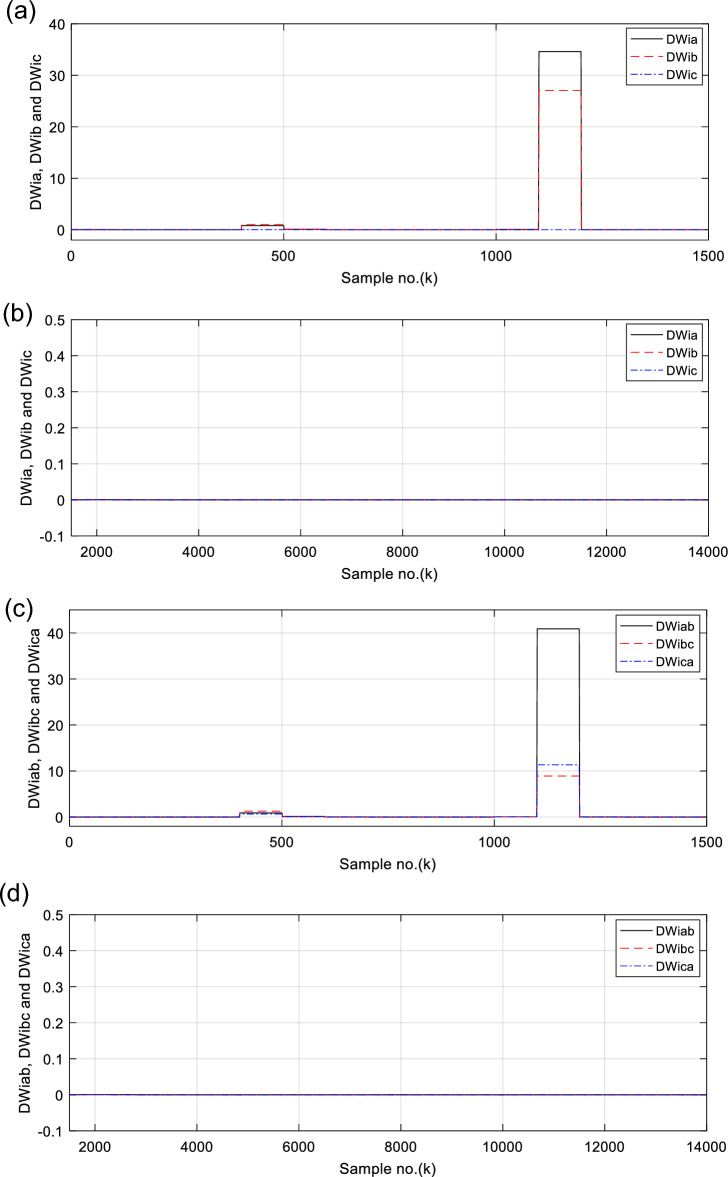
(**a**) *DWi*_*a*_, *DWi*_*b*_, and *DWi*_*c*_ for case 1 (before the fault initiation and during the fault time). (**b**) *DWi*_*a*_, *DWi*_*b*_, and *DWi*_*c*_ for case 1 (after the fault clearance). (**c**) *DWi*_*ab*_, *DWi*_*bc*_, and *DWi*_*ca*_ for case 1 (before the fault initiation and during the fault time). (**d**) *DWi*_*ab*_, *DWi*_*bc*_, and *DWi*_*ca*_ for case 1 (after the fault clearance). (**a**–**d**) Durbin Watson* (DW)* factors of the current signals for case 1.

**Fig. 7:**
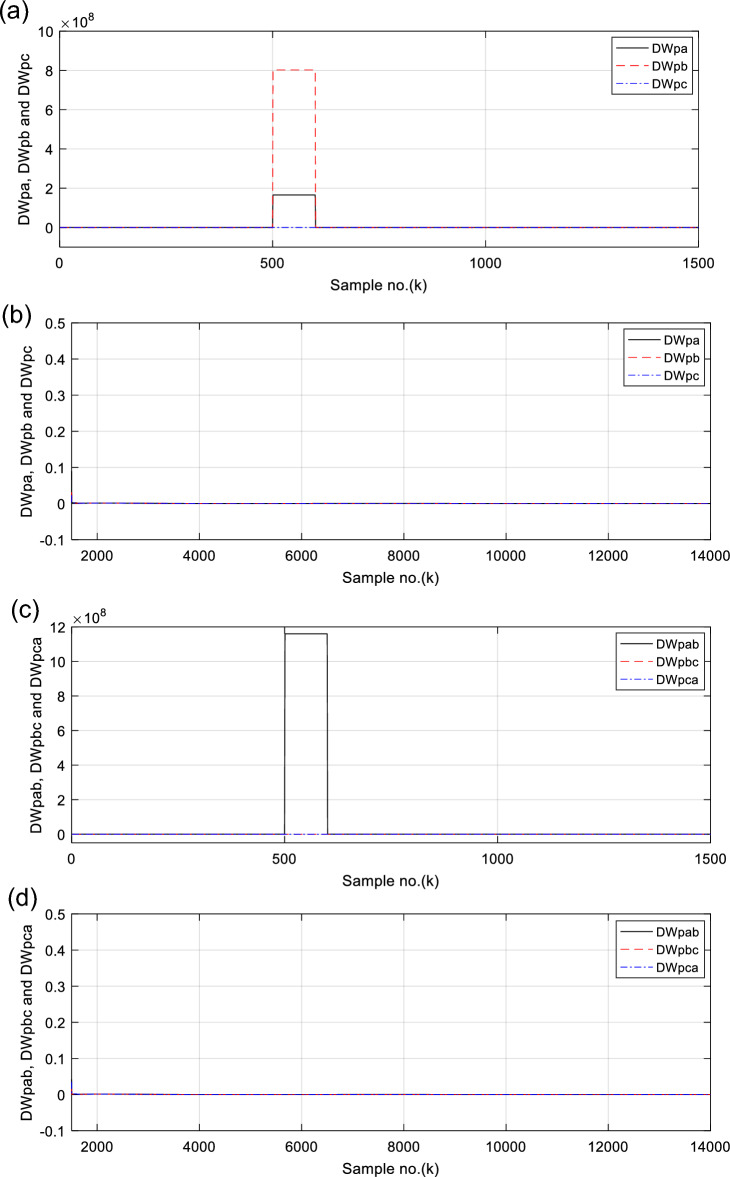
(**a**) *DWp*_*a*_, *DWp*_*b*_, and *DWp*_*c*_ for case 1 (before the fault initiation and during the fault time). (**b**) *DWp*_*a*_, *DWp*_*b*_, and *DWp*_*c*_ for case 1 (after the fault clearance). (**c**) *DWp*_*ab*_, *DWp*_*bc*_, and *DWp*_*ca*_ for case 1 (before the fault initiation and during the fault time). (**d**) *DWp*_*ab*_, *DWp*_*bc*_, and *DWp*_*ca*_ for case 1 (after the fault clearance). (**a**–**d**) Durbin Watson (*DW*) factors of the active power signals for case 1.

**Fig. 8:**
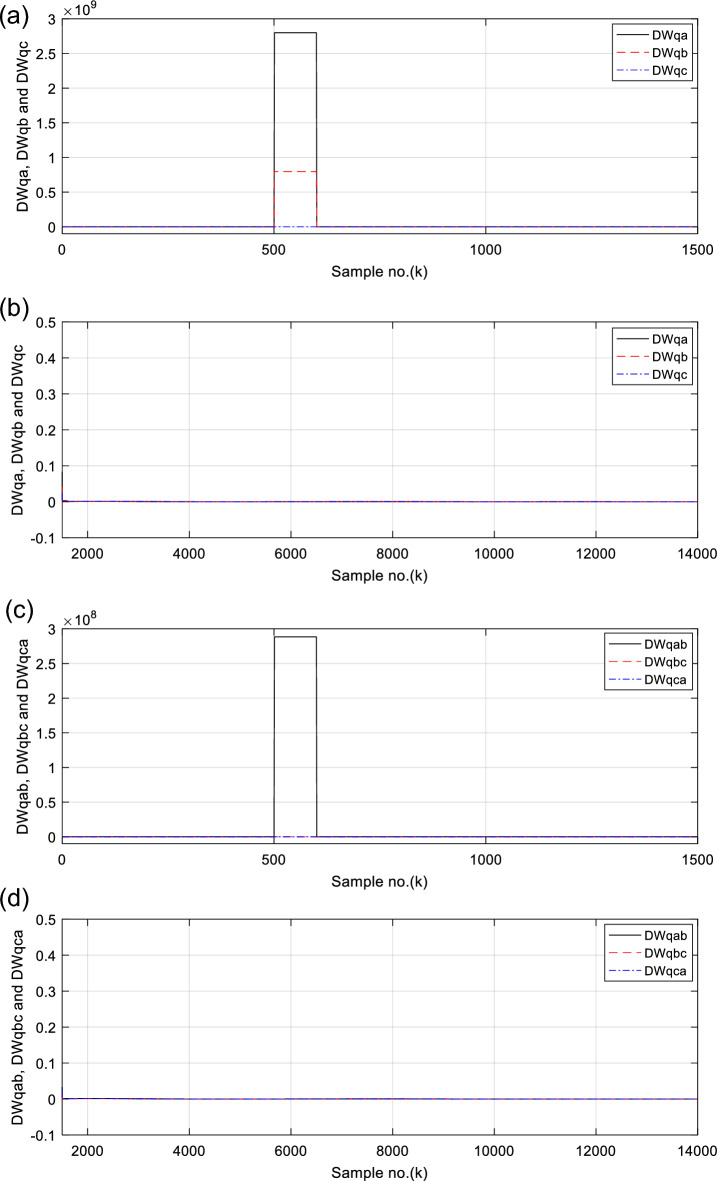
(**a**) *DWq*_*a*_, *DWq*_*b*_, and *DWq*_*c*_ for case 1 (before the fault initiation and during the fault time). (**b**) *DWq*_*a*_, *DWq*_*b*_, and *DWq*_*c*_ for case 1 (after the fault clearance). (**c**) *DWq*_*ab*_, *DWq*_*bc*_, and *DWq*_*ca*_ for case 1 (before the fault initiation and during the fault time). (**d**) *DWq*_*ab*_, *DWq*_*bc*_, and *DWq*_*ca*_ for case 1 (after the fault clearance). (**a**–**d**) Durbin Watson (*DW*) factors of the reactive power signals for case 1.

**Fig. 9:**
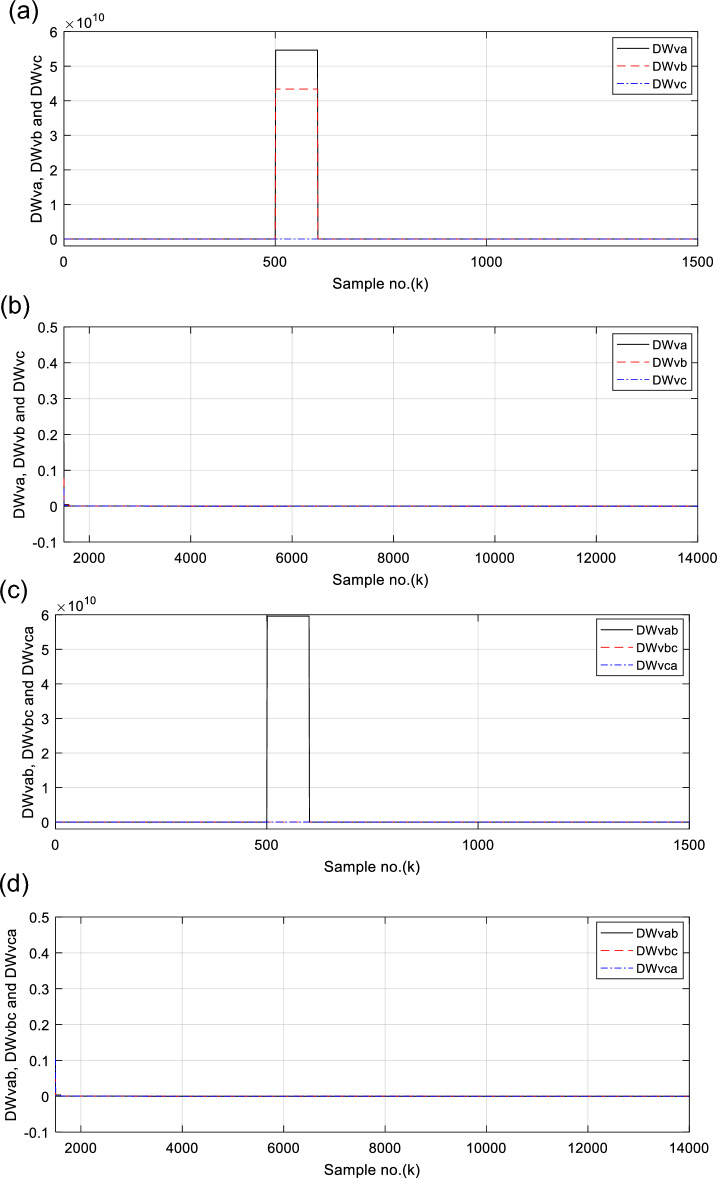
(**a**) *DWv*_*a*_, *DWv*_*b*_, and *DWv*_*c*_ for case 1 (before the fault initiation and during the fault time). (**b**) *DWv*_*a*_, *DWv*_*b*_, and *DWv*_*c*_ for case 1 (after the fault clearance). (**c**) *DWv*_*ab*_, *DWv*_*ba*_, and *DWv*_*ca*_ for case 1 (before the fault initiation and during the fault time). (**d**) *DWv*_*ab*_, *DWv*_*ba*_, and *DWv*_*ca*_ for case 1 (after the fault clearance). (**a**–**d**) Durbin Watson (*DW*) factors of the voltage signals for case 1.

**Fig. 10:**
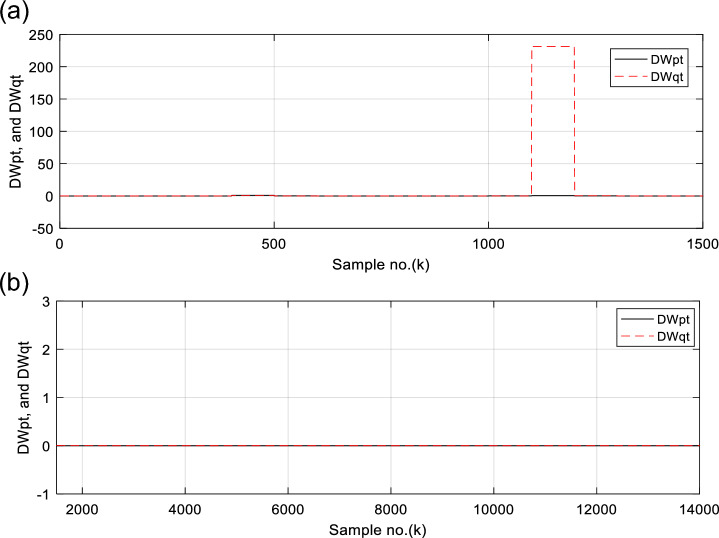
(**a**) *DWp*_*t*_, and *DWq*_*t*_ for case 1 (before the fault initiation and during the fault time). (**b**) *DWp*_*t*_, and *DWq*_*t*_ for case 1 (after the fault clearance). (**a**–**b**) Durbin Watson (*DW*) factors of the total active and reactive powers for case 1.

Figures 6, 7, 8, 9, 10 illustrate the twenty-six DW indices of the electrical signals for case study 1. The indices indicate the state of synchronous operation of the SG following the fault clearance. Figure 6a–d depict the DW factors for the current signals, Fig. 7a–d show the DW factors for the active power signals, Fig. 8a–d display the DW factors for the reactive power signals, Fig. 9a–d offer the DW factors for the voltage signals, and Fig. 10a, b present the DW factors for the total active and reactive powers for case study 1. Before fault occurrence and after fault clearing, the quantities of the following twenty-six *DW* indices are nearly zero: *DWi*_*a*_*, DWi*_*b*_*, DWi*_*c*_*, DWi*_*ab*_*, DWi*_*bc*_*, DWi*_*ca*_*, DWp*_*a*_*, DWp*_*b,*_* DWp*_*c*_*, DWp*_*ab*_*, DWp*_*bc,*_* DWp*_*ca*_*, DWq*_*a,*_* DWq*_*b*_*, DWq*_*c*_*, DWq*_*ab,*_* DWq*_*bc*_*, DWq*_*ca*_*, DWv*_*a*_*, DWv*_*b*_*, DWv*_*c*_*, DWv*_*ab*_*, DWv*_*ba*_*, DWv*_*ca*_* DWp*_*t*_, and *DWq*_*t*_. Figure 6a, b illustrate *DWi*_*a*_*, DWi*_*b*_*,* and *DWi*_*c*_, and Fig. 6c, d show *DWi*_*ab*_*, DWi*_*bc*_*,* and *DWi*_*ca*_ for case 1. Figure 7a, b demonstrate *DWp*_*a*_*, DWp*_*b*_, and *DWp*_*c*_, and Fig. 7c, d depict *DWp*_*ab*_*, DWp*_*bc,*_ and *DWp*_*ca*_ for case 1. Figure 8a, b present *DWq*_*a,*_* DWq*_*b*_*,* and *DWq*_*c*_, and Fig. 8c, d describe *DWq*_*ab,*_* DWq*_*bc*_*,* and *DWq*_*ca*_ for case 1. Figure 9a, b introduce *DWv*_*a*_*, DWv*_*b*_*,* and *DWv*_*c*_, and Fig. 9c, d offer *DWv*_*ab*_*, DWv*_*ba*_*,* and *DWv*_*ca*_ for case 1. Figure 10a, b manifest *DWp*_*t*_, and *DWq*_*t*_. These values affirm the synchronization and normal operating conditions of the SG before the fault commencement and after the fault clearance.

For case 1, the following observations can be noticed during the fault time span:

(III) As depicted in Fig. 3a, b, the voltages of the two faulty phases (‘*A*’ and ‘*B*’) drop to roughly zero, while a transient is existent in their currents, as illustrated in Fig. 3c, d

(IV) As depicted in Fig. 4a, b, the phase active and reactive powers of the two faulty phases (‘*A*’ and ‘*B*’) collapse, respectively.

(V) Momentary variations occur in the curves of the phase voltage, active and reactive powers of the healthy phase ‘*C*’.

(VI) As a result, the total active power vanishes, while the total reactive power changes rapidly, as presented in Fig. 4c

(VII) The numerical values of the PR factor transfer from + 1.0 to −1.0, as shown in Fig. 4d

(VIII) The numerical values of the PFA, PF, load angles become unstable and changes quickly, as presented in Fig. 5a–c, respectively,

(IX) The values of the DW factors of the currents, phase active and reactive powers, voltages, and the total active and reactive powers change suddenly at the fault initiation and get unequal, as demonstrated in Figs. 6, 7, 8, 9, and 10, respectively, and

(X) The DW factors of the three phase voltages and currents can discover the abnormal conditions, categorize the fault type (*DLG*), and discriminate the two faulty phases (*A* and *B*), as illustrated in Figs. 6 and 9, respectively.

For case 1, the following points can be observed after fault clearing:

1. The three phase currents, active and reactive powers oscillate lightly; these quantities come back to their original values after few seconds of the fault clearance, as illustrated in Fig. 3c, d and 4a, b, respectively,
2. The quantities of the total active power are higher than zero, as introduced in Fig. 4c
3. The values of PR factor are + 1.0, as depicted in Fig. 4d
4. The PFA has acceptable fluctuations between the two values of 19° and 36°, as given in Fig. 5a. Hence, the PF ranges between the two values of 0.95 and 0.81 after fault clearing, as presented in Fig. 5b
5. The load angles (*δ*_*a*_, *δ*_*b*_ and *δ*_*c*_) range between 21.3 and 37.8°, as demonstrated in Fig. 5c
6. All DW factors are fixed and balanced, where their values are close to zero, as shown in Figs. 6, 7, 8, 9, and 10
7. As illustrated in Fig. 4c, the sample index ‘*J*’, at which *P*(*J*) = *Q*(*J*), is not present. Besides, the zero-crossing points ‘*Z*_1_ and *Z*_2_’ at which *P*(*Z*_1_) < 0 and *P*(*Z*_2_) > 0 are not verified after fault clearing, and
8. The quantitative findings assure that the SG under test is synchronous with the rest of the power grid.

From the given results, there are mildly swings for the three phase voltage, current and power waves after fault clearing. Furthermore, it is obvious that all Durbin Watson (*DW*) indices computed for the electrical variables are balanced and close to normal values before the fault onset and after fault clearance. As a consequence, the SG is synchronizing with the remaining power network.

### Out-of-step condition (δ_1_ = 21°)

**Fig. 11:**
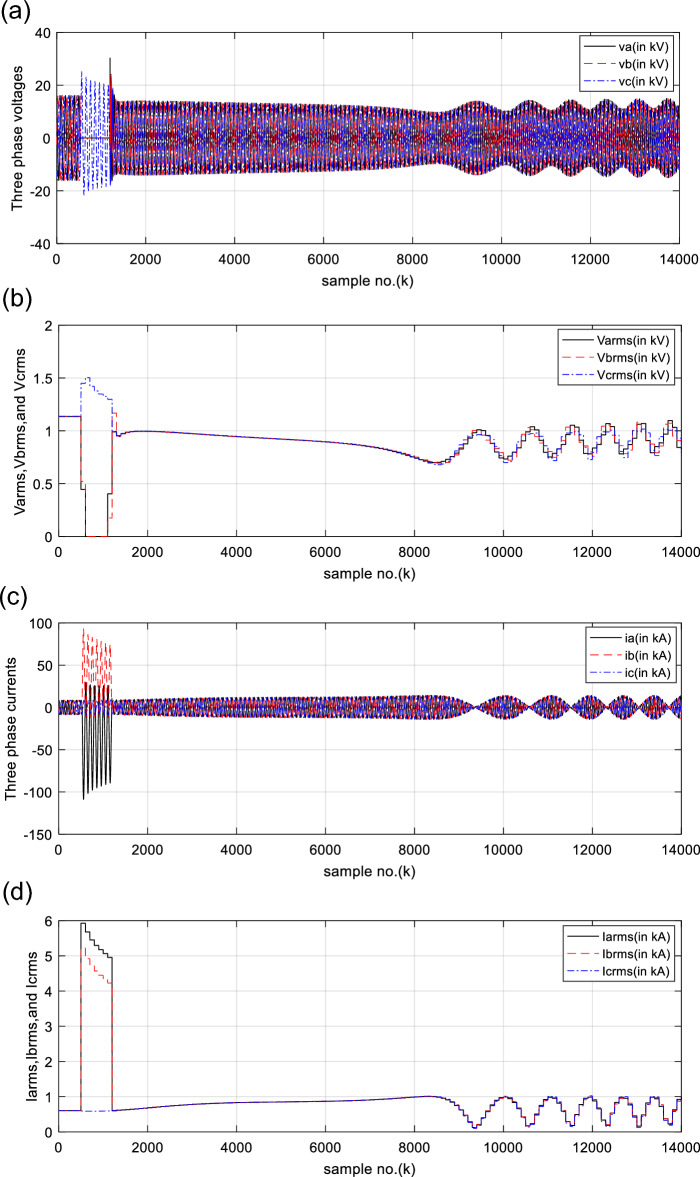
(**a**) Three-phase primary voltage waves taken at the SG load terminals. (**b**) RMS values of three-phase primary voltage waves taken at the SG load terminals. (**c**) Three-phase primary current waves taken at the SG load terminals. (**d**) RMS values of three-phase primary current waves taken at the SG load terminals. (**a**–**d**) Electrical waveforms for case 2.

**Fig. 12:**
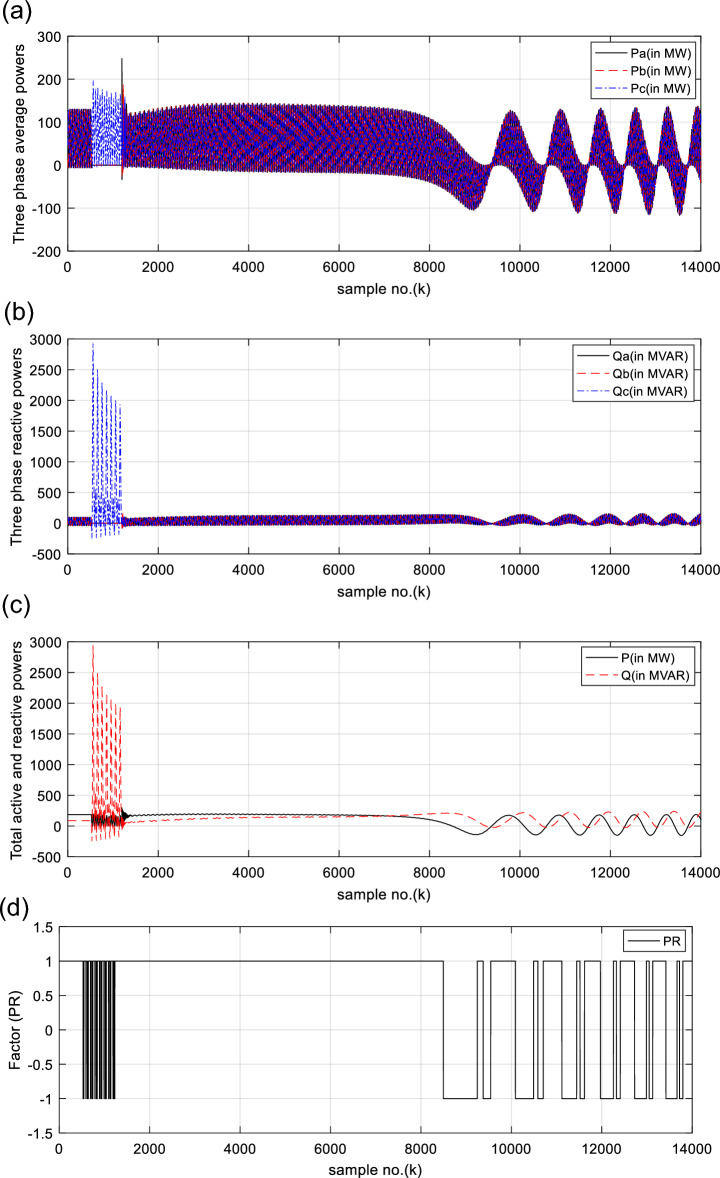
**a** Three-phase primary average power waves taken at the SG load terminals. (**b**) Three-phase primary reactive power waves taken at the SG load terminals. (**c**) Total primary average and reactive powers taken at the SG load terminals. (**d**) *PR* factor for case 2. (**a**–**d**) Electrical waveforms and *PR* factor for case 2.

In this instant, the SG load angle is *δ*_1_ = 21°, while the power system angle is *δ*_2_ = 0°. The system operating conditions during the time of DLG (A-B-G) fault are tabulated in Table 2. The obtained results emphasize that the SG connected to the power network operates under the influence of the OOS circumstances. The values of the pre-fault voltages, currents, active and reactive powers are constant, as seen in Figs. 11a–d, and 12a, b, respectively. Figure 11a, b introduce the three-phase primary voltage waves taken at SG load ends and their RMS values, respectively. Figure 11c, d present the three-phase primary current waves obtained at SG load ends and their RMS values, respectively.

The waveforms of the three-phase primary active and reactive powers generated at the SG load ends are depicted in Fig. 12a, b, respectively. Figure 12c shows the total primary active and reactive powers generated at the SG load ends, where their quantities are higher than zero after the fault clearance. All electrical waveforms are constant and symmetrical before the fault initiation, as exhibited in Figs. 11a–c, 12a, b. Whereas, they change extremely during the fault time, and severe swings appear in the waveforms after fault clearing. The total active and reactive powers swing sharply, as depicted in Fig. 12a–c. Also, the polarity of the total real power alters from the positive sign to the negative sign, and vice versa, as illustrated in Fig. 12c. This manifests that the SG is prone to a loss-of-synchronism condition with the remaining power grid. The PR factor for case 2 is depicted in Fig. 12d. It is clear that the PR values are + 1.0 before the fault occurrence, while its values vary from + 1.0 to −1.0, and vice versa, during the fault time and after the fault clearance, as shown in Fig. 12d. This factor asserts the OOS event for the SG.

**Fig. 13:**
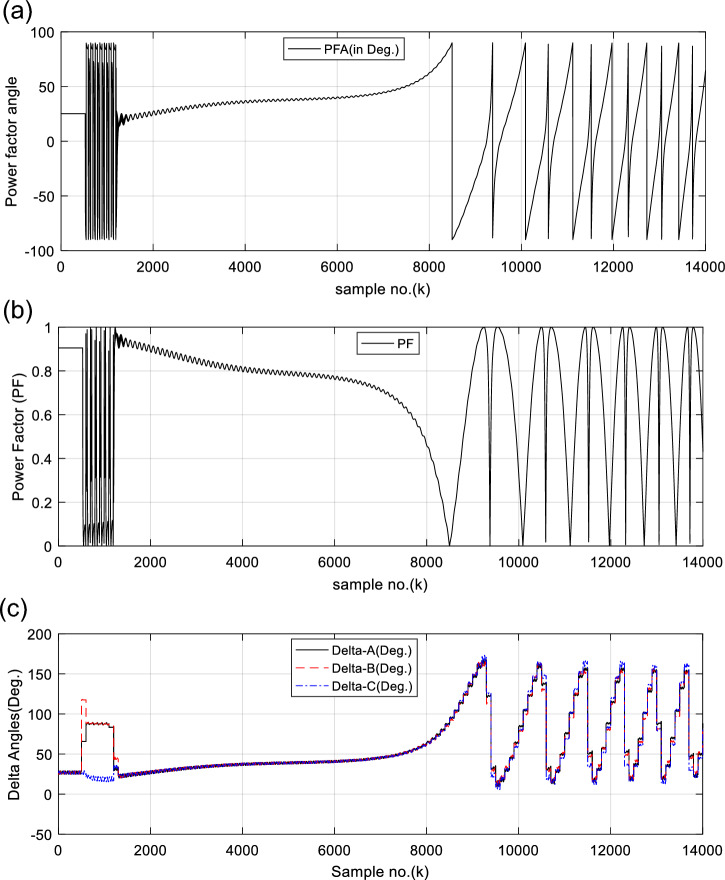
(**a**) Power factor angle (*PFA*) for case 2. (**b**) Power factor (*PF*) for case 2. (**c**) Computed power angles (*δ*_*s*_) for case 2. (**a**–**c**) *PFA*, *PF*, *Delta-A*, *Delta-B*, and *Delta-C* for case 2.

Before and during the fault time, the values of the electrical variables, the DW factors, and the indices derived from the electrical powers of case 2 are similar to those of case 1, as shown in Table 3. This means that all observations noted in case 1 are similar to those of case 2 before and during the fault time. Whereas, their quantities oscillate after the fault clearance. Thus, the quantitative findings indicate that the SG loses the synchronization with the remaining power network. Figure 13a and b present the power factor angle (*PFA*) and the power factor (*PF*) for case 2, respectively. The *PFA* is steady and equal to nearly 25° before the fault inception time, but it fluctuates greatly between + 90 and −90° during the fault period, as exhibited in Fig. 13a. Therefore, the PF is approximately 0.91 before the fault time, and it swings quickly between 0.0 and + 1.0 during the fault. Also, the PFA fluctuates sharply owing to the OOS situation after the fault clearing. Thus, the PFA varies in between + 90 and −90°, resulting in the PF ranges from 0.0 to + 1.0 after fault clearing, as presented in Fig. 13b. This means that the maximum deviation of the PFA is roughly 180^o^ after fault clearing, which assures the OOS event. The load angles (*δ*_*a*_, *δ*_*b*_ and *δ*_*c*_) computed for the three phases in the case of OOS are presented in Fig. 13c. It is clear that the load angles surpass 90°, and they fluctuate between 9.8 and 167.7° after fault clearing for case study 2, as shown in Fig. 13c.

**Fig. 14:**
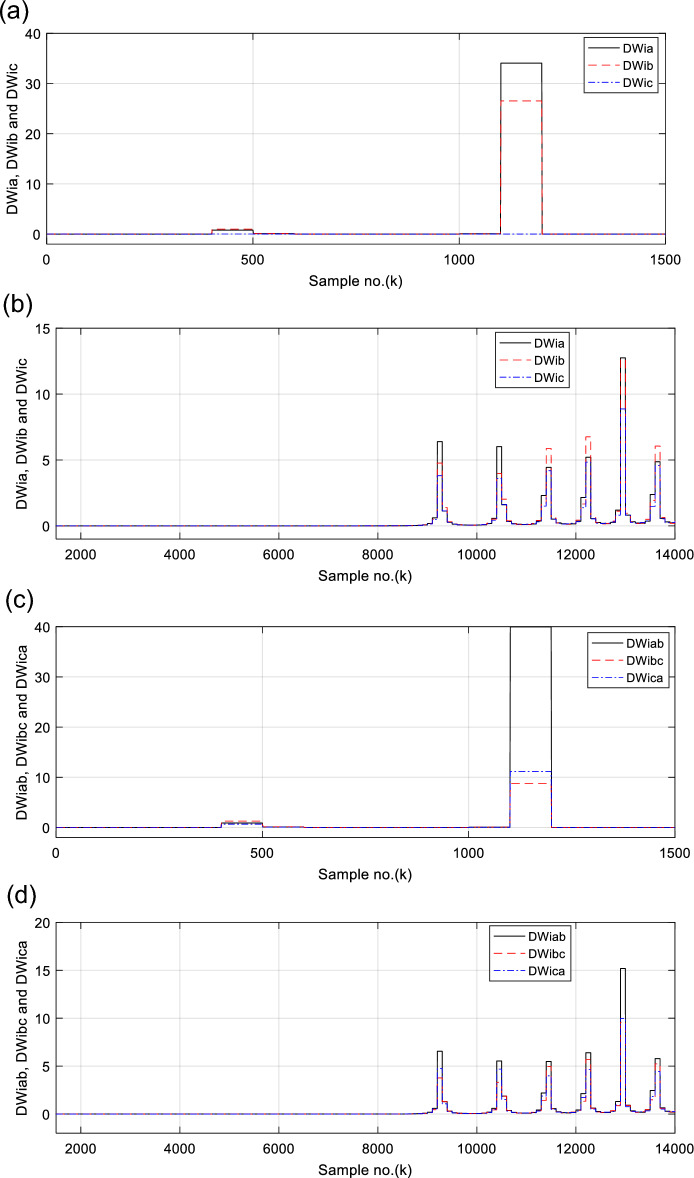
(**a**) *DWi*_*a*_, *DWi*_*b*_, and *DWi*_*c*_ for case 2 (before the fault initiation and during the fault time). (**b**) *DWi*_*a*_, *DWi*_*b*_, and *DWi*_*c*_ for case 2 (after the fault clearance). (**c**) *DWi*_*ab*_, *DWi*_*bc*_, and *DWi*_*ca*_ for case 2 (before the fault initiation and during the fault time, from sample index 1 to 1500). (**d**) *DWi*_*ab*_, *DWi*_*bc*_, and *DWi*_*ca*_ for case 2 (after the fault clearance, from sample index 1500 to 14,000). (**a**–**d**) Durbin Watson (*DW*) factors of the current signals for case 2.

**Fig. 15:**
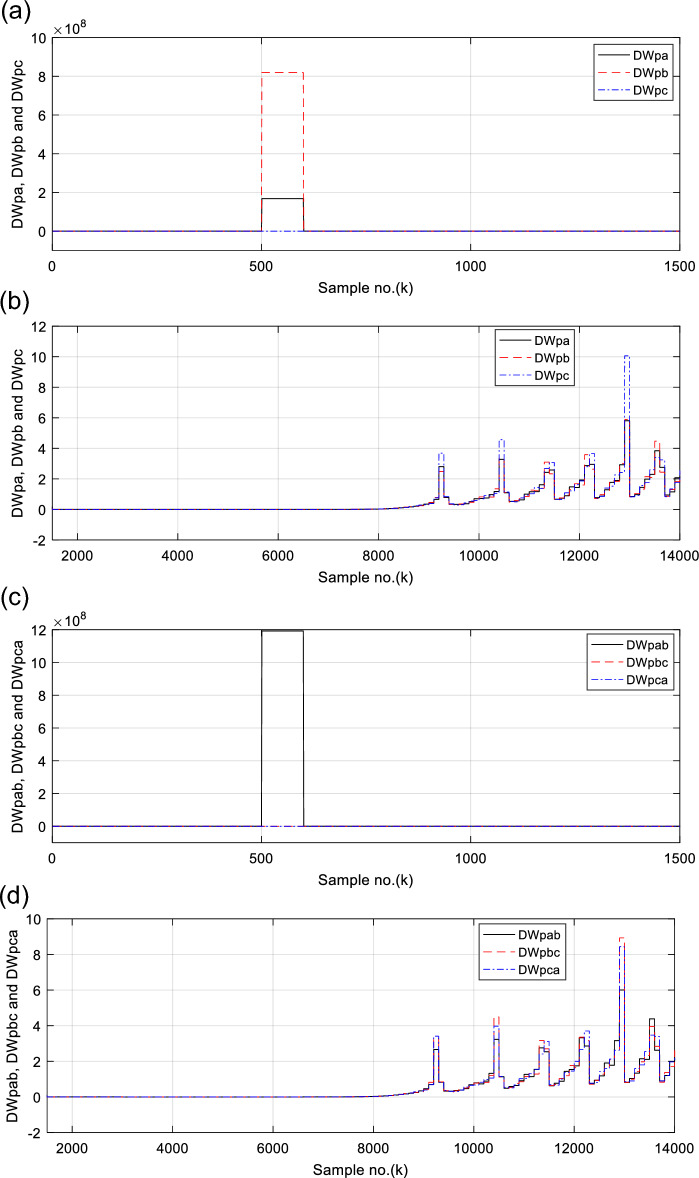
(**a**) *DWp*_*a*_*, DWp*_*b,*_ and *DWp*_*c*_ for case 2 (before the fault initiation and during the fault time, from sample index 1 to 1500). (**b**) *DWp*_*a*_, *DWp*_*b*_, and *DWp*_*c*_ for case 2 (after the fault clearance, from sample index 1500 to 14,000). (**c**) *DWp*_*ab*_, *DWp*_*bc*_, and *DWp*_*ca*_ for case 2 (before the fault initiation and during the fault time, from sample index 1 to 1500). (**d**) *DWp*_*ab*_, *DWp*_*bc*_, and *DWp*_*ca*_, for case 2 (after the fault clearance, from sample index 1500 to 14,000). (**a**–**d**) Durbin Watson* (DW)* factors of the active power signals for case 2.

**Fig. 16:**
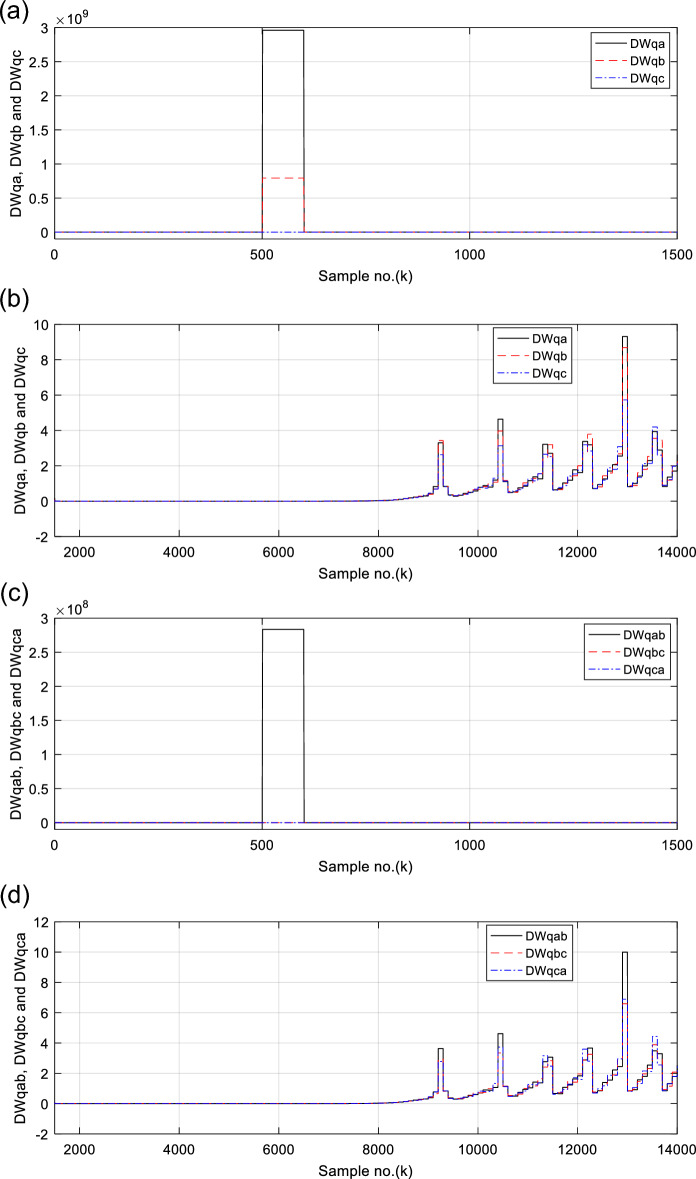
(**a**) *DWq*_*a*_, *DWq*_*b*_, and *DWq*_*c*_ for case 2 (before the fault initiation and during the fault time, from sample index 1 to 1500). (**b**) *DWq*_*a*_, *DWq*_*b*_, and *DWq*_*c*_ for case 2 (after the fault clearance, from sample index 1500 to 14,000). (**c**) *DWq*_*ab*_, *DWq*_*bc*_, and *DWq*_*ca*_ for case 2 (before the fault initiation and during the fault time, from sample index 1 to 1500). (**d**) *DWq*_*ab*_, *DWq*_*bc*_, and *DWq*_*ca*_ for case 2 (after the fault clearance, from sample index 1500 to 14,000). (**a**–**d**) Durbin Watson (*DW*) factors of the reactive power signals for case 2.

**Fig. 17:**
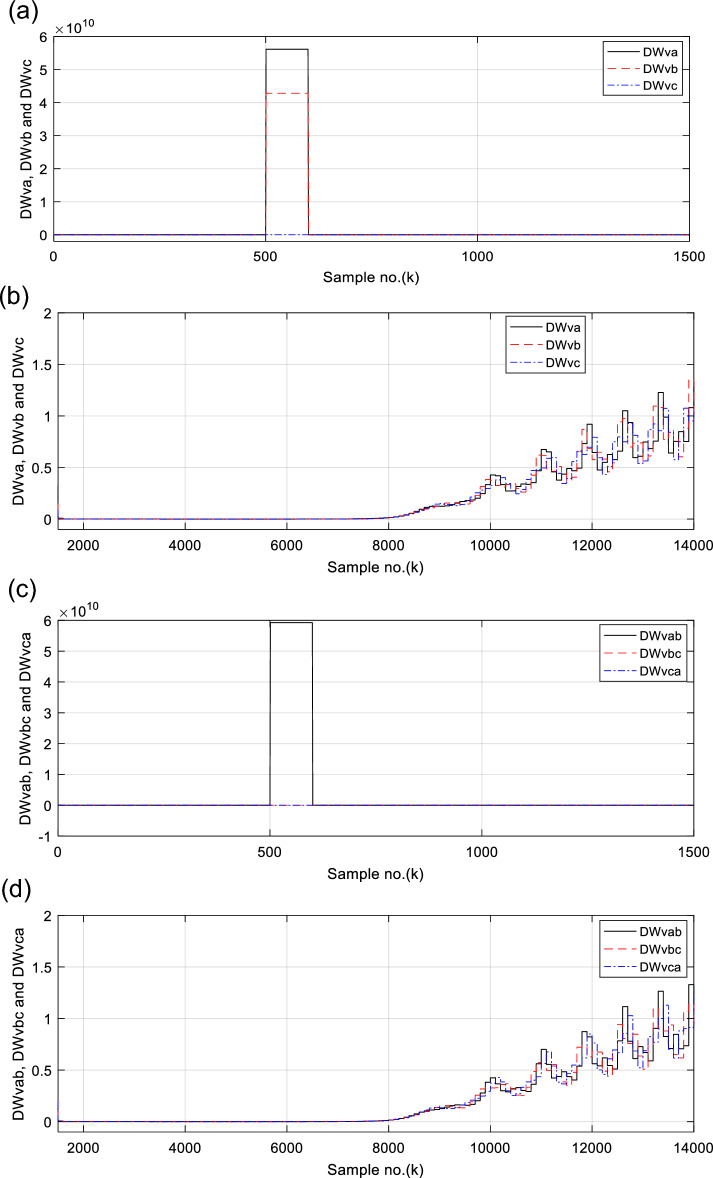
(**a**) *DWv*_*a*_, *DWv*_*b*_, and *DWv*_*c*_ for case 2 (before the fault initiation and during the fault time). (**b**) *DWv*_*a*_, *DWv*_*b*_, and *DWv*_*c*_ for case 2 (after the fault clearance). (**c**) *DWv*_*ab*_, *DWv*_*ba*_, and *DWv*_*ca*_ for case 2 (before the fault initiation and during the fault time). (**d**) *DWv*_*ab*_, *DWv*_*ba*_, and *DWv*_*ca*_ for case 2 (after the fault clearance). (**a**–**d**) Durbin Watson (*DW*) factors of the voltage signals for case 2.

**Fig. 18:**
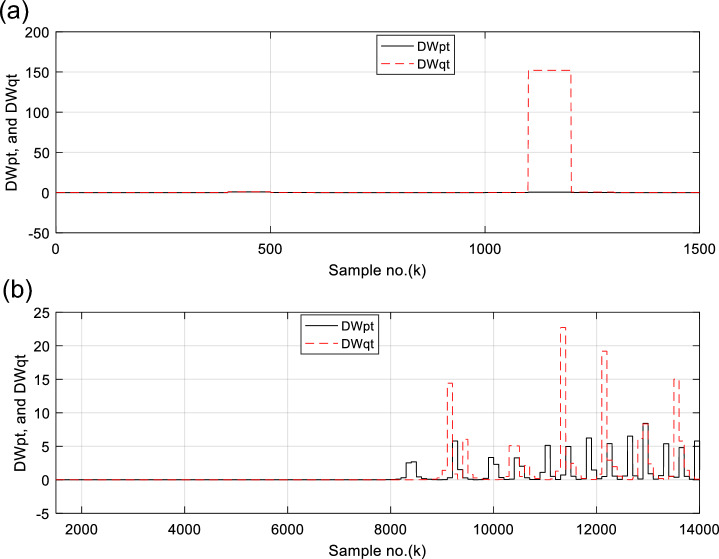
(**a**) *DWp*_*t*_, and *DWq*_*t*_ for case 2 (before the fault initiation and during the fault time). (**b**) *DWp*_*t*_, and *DWq*_*t*_ for case 2 (after the fault clearance). (**a**–**b**) Durbin Watson (*DW*) factors of the total active and reactive powers for case 2.

For case 2, the twenty-six DW indices of the electrical waves are presented in Figs. 14, 15, 16, 17, 18. The indices reveal the state of asynchronous operation of the SG after fault clearance. The DW indices for the current waves are shown in Fig. 14a–d for case 2. Figure 15a–d depict the DW factors of the active power waveforms, and Fig. 16a–d display the DW factors of the reactive power waveforms for case 2.

Figure 17a–d show the DW indices for the voltage waves, and Fig. 18a, b introduce the DW indices for the total active and reactive powers for case 2. The pre-fault quantities of the following twenty-six *DW* factors are about zero: *DWi*_*a*_*, DWi*_*b*_*, DWi*_*c*_*, DWi*_*ab*_*, DWi*_*bc*_*, DWi*_*ca*_*, DWp*_*a*_*, DWp*_*b,*_* DWp*_*c*_*, DWp*_*ab*_*, DWp*_*bc,*_* DWp*_*ca*_*, DWq*_*a,*_* DWq*_*b*_*, DWq*_*c*_*, DWq*_*ab,*_* DWq*_*bc*_*, DWq*_*ca*_*, DWv*_*a*_*, DWv*_*b*_*, DWv*_*c*_*, DWv*_*ab*_*, DWv*_*ba*_*, DWv*_*ca*_* DWp*_*t*_, and *DWq*_*t*_*.*

The DW factors are shown in Figs. 14a–d, 15a–d, 16a–d, 17a–d, and 18a, b. These factors affirm the asynchronous and abnormal operating condition of the SG after fault clearing, since their quantities surpass the pre-determined threshold values of the DW (as given in Table 1). Table 4 lists the zero-crossing points of the total active power (*P*) for case 2. Table 5 presents the quantitative findings (obtained from the simulation results after fault clearance) for the electrical variables, DW factors, and the indices deduced from the electrical power analysis.

**Table 4:** The zero-crossing points of the total active power (*P*) for case 2.

Zero-crossing points of total active power (*P*)		T_Insability_ (Sec)	Slip frequency (Hz) (the frequency of the power swing)
Z_1_	Z_2_		
8496	9375		
10,090	10,586	0.2420	4.132
11,118	11,519	0.1866	5.359
11,970	12,323	0.1608	6.218
12,726	13,048	0.1450	6.896
13,419	13,719	0.1342	7.451
14,068	14,352	0.1266	7.898

**Table 5:** The quantitative findings of the electrical variables, DW factors, and the indices deduced from the electrical powers after fault clearing.

Parameter data	Case 1	Case 2											
Generator state	Synchronous operation state	Out-of-step state											
Operating power angles	*δ*_1_ = 20.5° *δ*_2_ = 0°	*δ*_1_ = 21° *δ*_2_ = 0°											
*j sample* (*P*(*j*)= *Q*(*j*)) > *0*	None	6999		9858		10,940		11,814		12,586		13,289	
*m sample P* (*Z*_1_) < 0 & *P* (*Z*_2_) > 0		8496	9375	10,090	10,586	11,118	11,519	11,970	12,323	12,726	13,048	13,419	13,719
Instability time (*T*_*st*_) (*in mSec*)		*T*_*st*_ = (10,586– 9375)*0.2 = 242.2		*T*_*st*_ = (11,519– 10,586)*0.2 = 186.6		*T*_*st*_ = (12,323– 11,519)*0.2 = 160.8		*T*_*st*_ = (13,048– 12,323)*0.2 = 145		*T*_*st*_ = (13,719– 13,048)*0.2 = 134.2		*T*_*st*_ = (14,352−13,719)*0.2 = 126.6	
*v*_*s*_ (*Z*) (*in kV*)		− 0.692	*v*_*s*_ (*Z*) (*in kV*)		− 0.692	*v*_*s*_ (*Z*) (*in kV*)		− 0.692	+ 1.382	− 0.481	+ 0.068	− 0.873	+ 1.299
*i*_*s*_ (*Z*) (*in kA*)		+ 0.999	+ 0.307	+ 1.290	+ 0.213	− 0.803	− 0.517	+ 0.953	− 0.559	− 1.269	+ 0.138	− 0.885	− 0.669
*P*_*s*_(*Z*) (*in MW*)		− 69.22	− 36.33	− 53.10	− 14.88	+ 69.26	− 70.38	+ 76.08	− 77.33	+ 61.10	+ 0.952	+ 77.30	− 87.00
*Q*_*s*_(*Z*) (*in* *MVAR*)		+ 69.26	+ 13.06	+ 23.28	+ 26.83	+ 99.29	+ 0.369	+ 83.78	+ 18.98	+ 29.89	+ 4.237	+ 96.69	− 4.268
*P*(*Z*) (*in MW*)		− 0.165	− 65.54	− 0.380	− 90.37	− 0.706	− 105.5	− 0.543	− 116.7	− 0.676	− 126.7	− 0.628	− 133.0
*Q*(*Z*) (*in MVAR*)		+ 207.5	− 11.12	+ 215.7	+ 2.499	+ 221.91	+ 15.87	+ 229.8	+ 25.73	+ 233.5	+ 35.15	+ 238.2	+ 43.89
*PFA*(*Z*) (*in Deg*)		− 89.9°	^+^ 9.63°	− 89.89	− 1.58	− 89.8	− 8.55	− 89.86	− 12.43	− 89.83	− 15.50	− 89.84	− 18.27
*PF*(*Z*)		0.000	0.985	0.001	0.999	0.003	0.988	0.002	0.976	0.003	0.963	0.002	0.949
*δ*_*s*_ (*Z*) (*inDeg*)		86.4°	163°	83.87°	160.8°	102.3°	152.3°	89.0°	152.4°	102.2°	155°	105°	150°
*PR*(*Z*)		− 1.0	− 1.0	− 1.0	− 1.0	− 1.0	− 1.0	− 1.0	− 1.0	− 1.0	− 1.0	− 1.0	− 1.0
*DWi*_*s*_ (*Z*)		0.008	6.389	0.062	6.010	0.143	4.436	0.154	5.206	0.266	12.75	0.362	4.864
*DWi*_*sx*_(*Z*)		0.008	6.547	0.062	5.537	0.163	5.485	0.165	6.399	0.319	15.18	0.443	5.789
*DWp*_*s*_(*Z*)		0.106	2.801	0.719	3.280	1.168	2.580	1.435	2.951	1.892	5.803	2.296	2.752
*DWp*_*sx*_(*Z*)		0.111	2.664	0.790	3.229	1.139	2.539	1.538	2.859	1.78	6.004	2.109	2.621
*DWq*_*s*_(*Z*)		0.104	3.299	0.781	4.635	1.379	2.703	1.774	3.185	2.063	9.315	2.295	2.891
*DWq*_*sx*_(*Z*)		0.100	3.632	0.698	4.618	1.417	3.063	1.623	3.663	2.217	9.994	2.542	3.295
*DWv*_*sx*_(*Z*)		0.057	0.159	0.425	0.319	0.555	0.484	0.822	0.619	0.779	0.727	0.825	0.846
*DWv*_*s*_ (*Z*)		0.055	0.152	0.427	0.274	0.651	0.435	0.919	0.549	0.936	0.608	0.988	0.753
*DWpt*(*Z*)		2.678	5.788	2.292	3.262	0.504	4.963	1.456	5.388	0.580	8.414	0.498	4.794
*DWqt*(*Z*)		0.000	0.860	0.005	5.056	0.1237	1.558	0.046	2.916	0.2944	8.341	0.513	5.783

For case study 2, the time intervals of the sudden changes in the DW factors after fault clearance are given in Table 6. Furthermore, maximum quantities of the DW factors after fault clearance for case 2 are tabulated in Table 7, where the quantities of the twenty-six factors exceed 1.0.

**Table 6:** The time intervals of the maximum sudden changes in the DW factors after fault clearance for case 2.

DWi_s_	DWv_s_ DWv_sx_	DWpt	DWqt
DWp_s_			
DWq_s_			
DWi_sx_			
DWp_sx_			
DWq_sx_			
9200–9300	10,000–10,100	8400–8500	9100–9200
		9200–9300	9400–9500
10,400–10,500	11,000–11,100	9900–10,000	10,300–10,400
		10,400–10,500	10,600–10,700
11,400–11,500	11,900–12,000	11,000–11,100	11,300–11,400
		11,400–11,500	11,500–11,600
12,200–12,300	12,600–12,700	11,800–11,900	12,100–12,200
		12,200–12,300	12,800–12,900
12,900–13,000	13,300–13,400	12,600–12,700	12,900–13,000
		12,900–13,000	13,500–13,600
13,600–13,700	13,900–14,000	13,400–13,500	13,600–13,700
		13,600–13,700	13,700–13,800

**Table 7:** Maximum quantities of the DW factors after fault clearance for case 2.

Parameter	Maximum values of DW factors after fault clearing for case 2					
DW factors of currents	DWi_a_	DWi_b_	DWi_c_	DWi_ab_	DWi_bc_	DWi_ca_
	12.75	12.61	8.875	15.187	9.581	9.976
DW factors of voltages	DWv_a_	DWv_b_	DWv_c_	DWv_ab_	DWv_bc_	DWv_ca_
	1.227	1.3579	1.075	1.329	1.151	1.129
DW factors of active powers	DWp_a_	DWp_b_	DWp_c_	DWp_ab_	DWp_bc_	DWp_ca_
	5.804	5.884	10.063	6.00	8.931	8.436
DW factors of reactive powers	DWq_a_	DWq_b_	DWq_c_	DWq_ab_	DWq_bc_	DWq_ca_
	9.315	8.686	5.727	9.995	6.590	6.893
DW factors of total active and reactive powers	DWp_t_	DWq_t_				
	8.414	22.712				

It is evident that the pre-fault values of all DW factors are fixed and close to 0.0, their values increase suddenly at the fault starting, as illustrated in Figs. 14a–d, 15a–d, 16a–d, 17a–d, and 18a, b. Moreover, it is observed that all factors vary sharply at the same instant after fault clearing of the simulation time. Besides, these variations occur six times for the DW factors computed for the phase currents, phase voltages, and phase active and reactive powers, and they are existing twelve times for the DW factors calculated for the total active and reactive powers, as shown in Table 6.

For case 2, the following observations can be concluded after fault clearance:

1. The three phase voltages, currents, active and reactive powers fluctuate severely, and their magnitudes remain infinitely oscillated after fault clearance, as presented in Fig. 11a–d, 12a, and b, respectively
2. The quantities of the total active power swing between the positive and negative values after fault clearance, as depicted in Fig. 12c
3. The values of PR factor vary from + 1.0 to − 1.0, as introduced in Fig. 12d
4. The PFA has unacceptable sharp fluctuations between the two angles of + 90° and -90°, as illustrated in Fig. 13a. Hence, the PF is bounded between the two values of 0.0 and + 1.0, as demonstrated in Fig. 13b
5. The load angles (*δ*_*a*_, *δ*_*b*_ and *δ*_*c*_) are greater than 90°, and they oscillate between 9.8 and 167.7°, as shown in Fig. 13c
6. All DW factors of the electrical waves change suddenly in the instant of the unstable power swing, and their values are greater than the DW setting values, as demonstrated in Figs. 14, 15, 16, 17, and 18
7. As shown in Fig. 12c, the sample index ‘*J*’, at which *P*(*J*) = *Q*(*J*), is existent. Moreover, the zero-crossing points ‘*Z*_1_ and *Z*_2_’ at which *P*(*Z*_1_) < 0 and *P*(*Z*_2_) > 0 are verified after fault clearance. The first sample position of ‘*J*’ at which *P*(*J*) = *Q*(*J*) is 6999 after fault clearing, and the sample positions of ‘Z’ at which *P* (*Z*_1_) < 0 and *P*(*Z*_2_) > 0 are six indices in the following sequence: 9375, 10,586, 11,519, 12,323, 13,048 and 13,719, as included in Tables 5 and 6. Therefore, it is noticed that unstable power swings occur in the electrical grid, then the SG loses the synchronization with the grid. The quantitative findings given in Tables 5, 6, 7 and 8 confirm the generator OOS condition,
8. The instability time and the frequency rate of the power swings can be determined using the zero-crossing points of the total active power (*P*) for case study 2, as noted in Table 5, and
9. The quantitative findings confirms that the SG under test is not synchronized with the remaining power network.

**Table 8:** A comparison between the proposed strategy and the several impedance-based techniques for SG OOS detection.

Item of comparison	The proposed SG OOS protection based on electrical power analysis and Durbin Watson testing	The existing SG OOS protection based on impedance criteria
1. Required waves	It needs the three-phase voltage and current waveforms measured at the load terminals of the SG stator windings	
2. Sampling rate	It requires high sampling rate	
3. Algorithm operation (online/offline)	The algorithm functions online to predict and detect the machine OOS events. Also, it can monitor continually the machine state	
4. Protection fundamentals	The present algorithm is based on dual numerical methods using the electrical power analysis and DW statistic to determine the SG out-of-step events	The protection method is based on the impedance calculations to identify the SG out-of-step events^21,26,29,31,33,37,38^
5. Stable power swings (SPS)	The suggested and conventional methods are inactive during the presence of Stable Power Swings (SPS)	
6. Technique limitation	The suggested and conventional techniques have no limitation on network ratings and the actions of the power station controllers, since it depends on the OOS fundamentals	
7. Protection speed	The suggested and conventional techniques detect the OOS speedily	
	The protection speed is controllable using the data window criteria, where it can be selected to be a sub-cycle or a single cycle for computing the DW factors and the other indices obtained from the instantaneous powers	It is dependent on the RMS quantities of the phase voltage and current measurements, which lasts at least one cycle for computing the impedance^21^
	Because it only uses a local data, the time taken for data transfer and processing is greatly reduced	
8. Protection failure	The protection method is able to identify both the large disturbance and the OOS events	The impedance-based existing techniques are unable to identify the OOS events when the SG is under-excited^22^
9. OOS measure	The DW indices for the voltage and current signals behave as an estimator for the SG out-of-step detection. Therefore, these indices serve as a proper measure for the level of synchronization or OOS	The OOS estimator is not provided in many existing methods^21,31,38^
10. Protection settings	It can determine the most convenient settings (whether adaptive or stationary settings) of the DW factors computed for all variables and the derived indices according to the prevailing conditions of the SG and its connected power grid	It selects stationary settings of the impedance based on the RMS quantities of the phase voltage and current waves according to the prevailing conditions of the SG and its connected power grid^21,26,29,31,33,37,38^
		It can select various settings for the impedance and time delay^21,26,31,38^
11. Instability time/swing frequency	The swing frequency can be estimated with the developed technique based on the DW indices and electrical power analysis	Multiple existing techniques are unable to estimate the instability time and the swing frequency^1,2,5,21,38^
	Also, the instability time until the OOS initiation can be neatly estimated	
12. Early OOS detection	The protection approach can be used to predict and early detect the OOS circumstances	Several protection models do not endue adequate information on the symptoms and signs of the OOS circumstance, as these symptoms are invisible and serious^1,5,12,23^
13. Required fault data	Information about the fault clearing time and fault type are not required	Many protection methods based on the Equal Area Criteria (EAC) demand information about the fault clearing time and fault type to define the OOS events^9,29,33^
14. Multiple functions	The proposed scheme has been employed to perform a number of protection functions as follows:	Conventional relays detect only the OOS and fault events^21,26,29,31,33,36–38^
	Identification and assessment of OOS or loss-of-synchronism situations	
	Detection and assessment of voltage and current disturbances	
	Detection and assessment of voltage and current unbalances, and	
	Differentiation between asynchronous and synchronous running of the synchronous generator, as well as between healthy and faulty conditions	
15. Low-pass filter	The data window concept, used for computing the DW factors, performs the operational role of the digital low-pass filter	Additional low-pass filters are required for several methods^29,33,37^
16. Protection simplicity	The proposed algorithm is based on a simple application and less mathematical operations, and the adjustment of the DW settings is easy	Some methods are based on complicated applications or extensive mathematical solutions^36,38^
17. Protection precision	The proposed algorithm can recognize the system instability precisely with a convenient prediction time	There is a lack of precision for OOS anticipation/detection in some traditional methods, as well as the time estimated until instability occurrence^13,23^
18. Protection reliability	The protection method achieves reliable operation. It is fundamentally assessed by the failure absence of the protection operation	It verifies the reliability property for operation
	The method detects the stability condition and infers the instability time in order to make smarter decisions after anticipating the OOS	The remaining time until the OOS occurrence cannot be determined^21^
19. Protection security	The security can be boosted by extending the selected data package or the DW settings of the proposed algorithm	The security can be boosted by expanding the relay operation time delay and reducing the impedance setting/curve of the existing impedance relay^21,26,31,38^
20. Protection dependability	The dependability can be enhanced by reducing the selected data package or the DW settings of the proposed algorithm	The dependability can be enhanced by minimizing the relay operation time delay and stretching the impedance setting/curve of the existing impedance relay^21,26,31,38^
21. Protection sensitivity	The sensitivity for detecting OOS can be raised by decreasing the data package or the DW settings of the protection algorithm	The sensitivity for detecting OOS can be increased by widening the impedance setting/curve of the existing impedance relay
	The protection security, dependability, and sensitivity are controllable using the predetermined data package or the DW settings	The security, dependability, and sensitivity of the existing method are controllable using the settings of the impedance relay, along with the operation time delay of the impedance relay^21,26,31,38^
22. Protection redundancy	The redundancy of the suggested protection is great because it can integrate dual protection algorithms, where it can function many DW factors and other indices derived from the instantaneous powers to confirm the OOS events	There is a lack of a redundancy for OOS anticipation/detection with the impedance relay^32,34^
23. Application environment	The suggested approach can be used to protect different sizes of synchronous generators against OOS events in various arrangements and types of electrical networks, such as traditional, hybrid, and smart grids	Some existing techniques can be applied to protect different sizes of synchronous generators against OOS events in traditional, hybrid, and smart grids^21,26,29,31,33,36–38^

From the numerous tests and their generated results, it is seen that the electrical waves get more stable after the DLG fault clearance. This happens when the SG works at a load angle of lower than 20.5°, as long as the same fault conditions. Whereas, the SG reaches an unstable state when its load angle exceeds 20.5°, causing the electrical waves to continuously swing. Moreover, the operational performance of the proposed algorithm is highly reliable and precise due to its ability to anticipate and identify the OOS events. Furthermore, it has the capability to differentiate between synchronous and asynchronous operations accurately. As a result, the DW factors of all electrical signals, along with the factors derived from the power formulas, serve as a proper OOS detector. The detector is able to determine whether the SG state is asynchronous/unstable or synchronous/stable with the rest of the power network.

## The salient characteristics of the strategy

### Comparative analysis

Numerous simulation tests have been conducted on different power system configurations with varying levels of voltage and load power. These verifications have considered distinct fault and operating conditions, including fault time duration, shunt fault category, fault location, fault resistance, SG load angle, loading level, power factor, and different parameters for the components of each power system. The simulation results have demonstrated the effectiveness and robustness of the proposed protection strategy. Furthermore, the strategy has the capability to detect both the ten shunt faults and unstable power swings. The technique is robust because it is inactive during the conditions of stable power swing and normal operation. Furthermore, the results emphases that the proposed algorithm detects the OOS situations upon which the protective relay sends a tripping order to the SG circuit breakers, yet it would be silent in the synchronous and normal operating conditions.

Table 8 presents a comparison between the proposed strategy and existing impedance-based protections for anticipating and detecting the SG OOS incidences. The table involves a variety of protection characteristics for the proposed strategy.

### Salient contributions

The main contributions of this research for OOS detection are as follows:

1. This article presents a novel digital protection scheme for out-of-step (OOS) detection for synchronous generators (SGs) using both electrical power analysis and Durbin Watson (DW) statistic,
2. The Durbin Watson (DW) algorithm is able to:
  Specify the fault and the out-of-step (OOS) events,
  Discern between the healthy and faulty machine states,
  Discriminate between the synchronized and non-synchronized operations of the AC generator with the remaining power grid,
  Discern between the stable and unstable power swings,
  Measure the power swing frequency/rate,
  Differentiate between the symmetrical and unsymmetrical voltages,
  Discriminate between the symmetrical and unsymmetrical currents,
  Measure the extent of the voltage and current asymmetry, and
  Estimate the degree of intensity of the voltage and current faults/disturbances,
3. The Durbin Watson (DW) factors can be integrated with new other factors derived from electrical powers to increase the reliability and redundancy of the algorithm for out-of-step detection and to protect the generator stator windings against different fault events,
4. It has the ability to estimate both the frequency of the power swings and the instability time precisely,
5. It can integrate with distance relays to ensure protection security during power swing and to avoid incorrect operation during stable power swing. Thus, it is useful for blocking distance relays when the power swing occurs, and
6. If a fault occurs during power swing, it must be detected and cleared by the protection relay as quickly as possible. Since power swings and three‐phase external faults are both balanced phenomena with respect to the SG protection zone, discriminating between them is very challenging.

## Conclusions

A severe short-circuit current may lead to OOS of a few AC generators, or it may make many AC generators out-of-service, resulting in a wide-area blackout development. Therefore, the OOS protective relay is recommended for SG protection against the loss-of-synchronism situations. To detect the OOS event, the proposed algorithm requires the three-phase voltage and current waves taken at the ends of the protected SG stator windings. Then it uses mathematical operations for computing a group of Durbin Watson (DW) factors, as well as some indices deduced from the mathematical models of the active and reactive powers. During the OOS, these factors surpass predetermined settings, and the tendency of the electrical waves demonstrates their characteristic variation during the OOS phenomenon. The mathematical expressions of the DW factors and the other derived indices have been validated using ATP and MATLAB software applications for varying the SG load angles in a real power model. The protection method has discriminated between the synchronous and asynchronous running of the SG with the rest of the power system. The OOS phenomenon has been detected and declared before the second pole-slipping event. Consequently, it could issue a tripping signal for segregating the SG under asynchronous or abnormal operating conditions, while it would be inactive under synchronous and normal operating conditions. The simulation results signify that the developed algorithm is able to identify the unstable power swing events in the power system due to the changes in the SG power angles. Besides, it is simple, accurate, effective, fast, secure, dependable, and stable. The data window concept is useful to avoid the impact of ripples, harmonics, or DC components. Moreover, the protection response speed, sensitivity, security, and stability are controllable using both data window size and the DW settings. The DW statistic reacts in the case of nonlinearity of the electrical waves, which results from the unstable power swings following the transient phenomenon. Whereas, it is inactive and robust in the case of stable power swings and no-fault conditions. Moreover, the algorithm has proved that the DW index is a smart measure for discriminating the generator synchronous and asynchronous running, and it operates correctly for both situations of faults and loss-of-synchronism. As a result, this confirms the suitability and effectiveness of the protection strategy. It is also easily comprehensible and feasible for implementation. DW setting values can be selected within a restricted and tiny range, thereby obviating the need for any mathematical analysis to identify these settings. In addition, the results show that the threshold characteristic used in the proposed algorithm is not affected by a power system configuration, given an efficient and reliable relay operation. Furthermore, the proposed strategy has the ability to value the instability times and the frequency rate of the unstable power swings. The OOS protection scheme can utilize the DW algorithm to perform the following functions:

- Figuring out the normal and abnormal states of the SG,
- Identifying and assessing generator OOS circumstances,
- Discerning between the synchronized and non-synchronized modes of the SG,
- Determining and estimating the disturbance levels of the three-phase voltages and currents, and
- Measuring and appreciating the asymmetry rates of the three-phase voltages and currents.

## Supplementary Information

Supplementary Information.

## Data Availability

All data generated or analysed during this study are included in this published article [and its supplementary information files].
