# Supplementary material for: Out-of-step detection for synchronous generators using electrical power analysis and Durbin Watson testing
Source: Sci Rep. 2025 Jun 20;15:20113. doi: 10.1038/s41598-025-03350-8 (PMC12181336; doi:10.1038/s41598-025-03350-8)
Supplement: Supplementary file 1 — Supplementary Information. [file 41598_2025_3350_MOESM1_ESM.pdf]

Nomenclatures:

Symbols	Abbreviations
$v_s(k)$	Instantaneous values of phase voltage (in Volt) at sample index *'k'* measured at synchronous generator terminals of phase *'S',*
$i_s(k))$	Instantaneous values of phase current (in Amp) at sample index *'k'* measured at synchronous generator terminals, of phase *'S',*
$P_s(k)$	Instantaneous values of active power per phase (in Watt) at sample index *'k'* calculated at the load end of SG, for phase *'S',*
$P_x(k)$	Instantaneous values of active power per phase (in Watt) at sample index *'k'* calculated at the load end of SG, for phase *'X',*
$Q_s(k)$	Instantaneous values of reactive power per phase (in VAR) at sample index *'k'* calculated at the load end of SG, for phase *'S',*
$Q_x(k)$	instantaneous values of reactive power per phase (in VAR) at sample index *'k'* calculated at the load end of SG, for phase *'X',*
$P_T(k)$	Total active power at instant '*k'* used to perform actual work,
$Q_T(k)$	Total reactive power at instant '*k'* required to move magnetic energy between the phase conductors in a power system,
Z	Zero-crossing positions (Z_1 and Z_2),
PF	Power Factor of the system,
PFA	Power Factor Angle (in Deg.),
PR	A factor based on $Q_T(k)$ and $P_T(k)$, which its values +1.0 during normal operating conditions, while its values -1.0 during fault or out-of-step events,
T_{os}	The predetermined time span used to monitor the generator OOS. It is set to 0.5 Sec in this algorithm,
θS	Power factor angle (in Deg.) for '*S*' phase,
δS	Load angle (in Deg.) for '*S*' phase,
θ_0	The maximum phase angle permissible that lies in between 45° and 90°,
δ_0	The maximum load angle permissible located within 90° and 180°,
$g_s(k)$	Sampled phase signal *g(k)* measured at instant *k* measured from the instrument transformer for '*S*' phase,
$g_s(k\text{-}Ns)$	Sampled value of the phase signal *g(k)* measured at the instant *(k-Ns)* for '*S*' phase,
$g_{sx}(k)$	Sampled line signal $g_{sx}(k)$ measured at instant *k* measured from the instrument transformer for '*S*' and '*X*' phases,
$g_{sx}(k\text{-}Ns)$	Sampled value of the line signal $g_{sx}(k)$ measured at the instant *(k-Ns)* for '*S*' and '*X*' phases,
DWg_s	Durbin Watson factor computed between each two successive data windows, shifted from each other by one cycle, where the data window contains *N* samples of the instantaneous values of the electrical signal ($g_s(k)$)

	measured/calculated for '*S*' phase,
DWg_{sx}	Durbin Watson factor computed between each two successive data windows, shifted from each other by one cycle, where the data window contains *N* samples of the instantaneous values of the electrical signal ($g_{sx}(k)$) calculated for '*S*' and '*X*' phases,
Δ1, Δ2, Δ3, and *Δ4*	The Durbin Watson (DW) settings; they lie between the values 0.0 and 2.0,
S and *X*	Phase designation *A*, *B* or *C* for the three phases, they are dissimilar,
k	Sample index,
N_s	Number of samples per cycle used in the simulation, ($Ns = Tc /Ts$ and $Ns = Fs /Fc$ = 100 Samples/cycle),
N_w	Number of samples per data window ($N_w \leq N_s$), ($N_w = Ns$ is selected in the technique),
Tc	Cycle time period, (T_c = 20 *mSec*),
Fc	Fundamental frequency of one periodic cycle, (F_c = 50 *Hz*),
T_s *or h*	Sampling time interval, ($T_s = h$ = 0.2 *mSec*),
Fs	Sampling frequency rate, (F_s = 5 *kHz*),
ω	Angular velocity of the power system, $\omega = 2\pi f$, (rad/sec),
R_f	Fault resistance imposed from the fault point on *SG* stator winding to the ground point in case of the ground fault or inserted between the two faulted phases in case of the phase fault,
ATP	Alternative Transient Program,
t_f	The instant of the fault occurrence,
N_f	Sample index of the fault occurrence,
T_c	The instant of the fault clearance,
N_c	The sample index of the fault clearance,
tfd	The fault time interval in (Sec),
Nfd.	The number of samples through the fault time interval,
N_{sim}	Full number of samples per the simulation time,
SLGF	Single Line-to-Ground Fault,
DLGF	Double Line-to-Ground Fault,
DLF	Double Line Fault,
3LGF	Three Line-to-Ground Fault,
SG	Synchronous Generator,
V_{sRMS} and	RMS values of voltage and current signals, respectively, of the phase '*S*',

I_{sRMS}	
V_{sMAX} and I_{sMAX}	MAX values of voltage and current signals, respectively, of the phase '*S*',
V_{1Max}	The peak phase voltage of the synchronous generator,
V_{2Max}	The peak phase voltage of the power network,
F_{1op}	The operating frequency of the synchronous generator,
F_{2op}	Operating frequency of the power network,
δ_1	Operating power angle of the synchronous generator,
δ_2	Operating power angle of the power network,
R_n	Generator grounding impedance through the neutral point,
V_n	Nominal voltage of the synchronous generator,
I_n	Nominal current of the synchronous generator,
SLD	Single Line Diagram,
FD	Fault Detection,
FL	Fault Location,
TL	Transmission Line,
VT	Voltage Transformer,
CT	Current Transformer,
VTR	Voltage Transformer Ratio,
CTR	Current Transformer Ratio,
R_b	Current transformer burden,
R_{CT}	Current transformer secondary winding resistance,
R_{lead}	Lead resistance connected between the current transformer terminals and the burden,
BB	Busbar,
CB	Circuit Breaker,

$v_a(k)$, $v_b(k)$ and $v_c(k)$: The instantaneous values of three-phase voltage signals at sample index '*k*' measured at the load terminal of SG, for the phases *A*, *B* and *C*, respectively,

$i_a(k)$, $i_b(k)$ and $i_c(k)$: The instantaneous values of three-phase current signals at sample index '*k*' measured at the load terminal of SG, for the phases *A*, *B* and *C*, respectively,

$P_a(k)$, $P_b(k)$ and $P_c(k)$: The instantaneous values of three-phase active power signals at sample index '*k*' calculated at the

load terminal of SG, for the phases *A*, *B* and *C*, respectively,

$Q_a(k)$, $Q_b(k)$ and $Q_c(k)$: The instantaneous values of three-phase reactive power signals at sample index *'k'* calculated at the load terminal of SG, for the phases *A*, *B* and *C*, respectively,

$P_T(k)$, and $Q_T(k)$: The instantaneous values of total active and reactive power signals at sample index *'k'* calculated at the load terminal of SG, respectively,

DWv_a: The Durbin Watson factor calculated for the phase voltage wave (v_a),

DWv_b: The Durbin Watson factor calculated for the phase voltage wave(v_b),

DWv_c: The Durbin Watson factor calculated for the phase voltage wave (v_c),

DWi_a: The Durbin Watson factor calculated for the phase current wave (i_a),

DWi_b: The Durbin Watson factor calculated for the phase current wave (i_b),

DWi_c: The Durbin Watson factor calculated for the phase current wave (i_c),

DWp_a: The Durbin Watson factor calculated for the phase active power wave (P_a),

DWp_b: The Durbin Watson factor calculated for the phase active power wave (P_b),

DWp_c: The Durbin Watson factor calculated for the phase active power wave (P_c),

DWq_a: The Durbin Watson factor calculated for the phase reactive power wave (Q_a),

DWq_b: The Durbin Watson factor calculated for the phase reactive power wave (Q_b),

DWq_c: The Durbin Watson factor calculated for the phase reactive power wave (Q_c),

DWp_t: The Durbin Watson factor calculated for the total average power wave (P_T),

DWq_t: The Durbin Watson factor calculated for the total reactive power wave (Q_T),

DWv_{ab}: The Durbin Watson factor calculated for the line voltage wave (v_{ab}),

DWv_{bc}: The Durbin Watson factor calculated for the line voltage wave (v_{bc}),

DWv_{ca}: The Durbin Watson factor calculated for the line voltage wave (v_{ca}),

DWi_{ab}: The Durbin Watson factor calculated for the line current wave (i_{ab}),

DWi_{bc}: The Durbin Watson factor calculated for the line current wave (i_{bc}),

DWi_{ca}: The Durbin Watson factor calculated for the line current wave (i_{ca}),

DWp_{ab}: The Durbin Watson factor calculated for the difference of active powers (P_a - P_b),

DWp_{bc}: The Durbin Watson factor calculated for the difference of active powers ($P_b – P_c$),

DWp_{ca}: The Durbin Watson factor calculated for the difference of active powers ($P_c – P_a$),

DWq_{ab}: The Durbin Watson factor calculated for the difference of reactive powers (Q_a - Q_b),

DWq_{bc}: The Durbin Watson factor calculated for the difference of reactive powers ($Q_b – Q_c$),

DWq_{ca}: The Durbin Watson factor calculated for the difference of reactive powers (Q_c- Q_a).

Appendix 1: The parameters' data of the power system components

**Parameters of power system components**	**Data**
**<u>Synchronous generator (Sending end):</u>**	
Rated Volt-ampere	*320 MVA*
Rated line voltage	*19.57 kV*
Voltage phasor angle	21^0
Rated frequency	*50 Hz*
Number of poles	*2*
Neutral grounding impedance (R_n)	*0.77 Ω*
**<u>Power grid (Receiving end):</u>**	
Nominal line voltage	*500kV*
Voltage phasor angle	0^0
Nominal frequency	*50 Hz*
Volt-ampere short circuit	*25 GVA* ($i_{s.c}$ *= 10 kA)*
**<u>Main Transformer:</u>**	
Rated Volt-ampere	*340 MVA*
Transformation voltage ratio	*19.57 kV /500 kV*
Connection primary/secondary	*Delta/Star earthed neutral*
Primary winding impedance (Z_p)	*0.0027 + j0.184 Ω*
Secondary winding impedance (Z_s)	*0.7708 + j 61.8 Ω.*
Vector group	*YNd1*
Z%	*15%*

**<u>Transmission Lines:</u>**<br><br>Positive sequence R<br><br>Zero sequence R<br><br>Positive sequence XL<br><br>Zero sequence XL<br><br>Positive sequence 1/Xc<br><br>Zero sequence 1/Xc<br><br>Transmission line long (Km)	 <br>*0.0217 Ω /km*<br><br>*0.247 Ω /km*<br><br>*0.302 Ω /km*<br><br>*0.91 Ω /km*<br><br>*3.96 µ℧ /km*<br><br>*2.94 µ℧ /km*<br><br>*200 Km*
**<u>Electrical Load 1:</u>**	13 + j8 Ω at line voltage *19.57 kV*
**<u>Electrical Load 2:</u>**	8.5 + j5.26 Ω at line voltage *500 kV*
**<u>Current Transformers:</u>**<br><br>CTR<br><br>Rated burden<br><br>Class	 <br>*12000/1 A*<br><br>*30 VA*<br><br>*5p20*
**<u>Voltage Transformers:</u>**<br><br>VTR<br><br>Rated burden<br><br>Class	 <br>*11000/100V*<br><br>*35 VA*<br><br>*0.5*
